# Development and Physico-Chemical and Antibacterial Characterization of Chromium-Doped Hydroxyapatite in a Chitosan Matrix Coating

**DOI:** 10.3390/polym17192633

**Published:** 2025-09-29

**Authors:** Daniela Predoi, Carmen Steluta Ciobanu, Simona Liliana Iconaru, Roxana Alexandra Petre, Krzysztof Rokosz, Steinar Raaen, Mihai Valentin Predoi

**Affiliations:** 1 National Institute of Materials Physics, Atomistilor Street, No. 405A, P.O. Box MG 07, 077125 Magurele, Romania; ciobanucs@gmail.com (C.S.C.); simonaiconaru@gmail.com (S.L.I.); 2 Department of Mechanics, University Politehnica of Bucharest, BN 002, 313 Splaiul Independentei, Sector 6, 060042 Bucharest, Romania; petre.roxana.alexandra@gmail.com; 3 Faculty of Electronics and Computer Science, Koszalin University of Technology, Śniadeckich 2, PL 75-453 Koszalin, Poland; rokosz@tu.koszalin.pl; 4 Department of Physics, Norwegian University of Science and Technology (NTNU), Realfagbygget E3-124 Høgskoleringen 5, NO 7491 Trondheim, Norway; steinar.raaen@ntnu.no

**Keywords:** chromium-doped hydroxyapatite, chitosan, ultrasound studies, physicochemical properties, biocompatibility, antimicrobial activity

## Abstract

Chromium-doped hydroxyapatite (7CrHAp) and chromium-doped hydroxyapatite in chitosan matrix (7CrHAp-CH) coatings were synthesized in order to address the need for biomaterials with improved physico-chemical and biological properties for biomedical applications. Both chromium-doped hydroxyapatite (7CrHAp) and chromium-doped hydroxyapatite in chitosan matrix (7CrHAp-CH) coatings could represent promising materials for biomedical applications due to their superior properties. This study aims to evaluate the physico-chemical and in vitro biological properties of 7CrHAp and 7CrHAp-CH coatings to determine the impact of chitosan incorporation on the physico-chemical and biological features. The results reported in this study indicate that addition of chitosan improves surface uniformity and biological properties, highlighting their potential for uses in biomedical applications. In this study, coatings of chromium-doped hydroxyapatite (7CrHAp, with x_Cr_ = 0.07) and its composite variant embedded in a chitosan matrix (7CrHAp-CH) were systematically analyzed using a suite of characterization techniques: X-ray diffraction (XRD), scanning electron microscopy (SEM), energy-dispersive X-ray spectroscopy (EDX), Fourier-transform infrared spectroscopy (FTIR), X-ray photoelectron spectroscopy (XPS), atomic force microscopy (AFM), and metallographic microscopy (MM). The results of the XRD analysis revealed that the average crystal size was 19.63 nm for 7CrHAp and 16.29 nm for 7CrHAp-CH, indicating a decrease in crystallite size upon CH incorporation. The films were synthesized via the dip coating method using stable suspensions, whose stability was assessed through ultrasonic measurements (double-distilled water serving as the reference medium). The values obtained for the stability parameter were 2.59·10^−6^ s^−1^ for 7CrHAp, 8.64·10^−7^ s^−1^ for 7CrHAp-CH, and 3.14·10^−7^ s^−1^ for chitosan (CH). These data underline that all samples are stable: CH is extremely stable, followed by 7CrHAp-CH (very stable) and 7CrHAp (stable). The in vitro biocompatibility of the 7CrHAp and 7CrHAp-CH coatings was evaluated with the aid of the MG63 cell line. The cytotoxic potential of these coatings towards MG63 cells was quantified using the MTT assay after 24 and 48 h of incubation. Our results highlight that both 7CrHAp and 7CrHAp-CH coatings exhibit high biocompatibility with MG63 cells, maintaining cell viability above 90% at both incubation times, thus supporting osteoblast-like cell proliferation. Furthermore, the antimicrobial efficacy of both 7CrHAp and 7CrHAp-CH samples was evaluated in vitro against the *Pseudomonas aeruginosa* 27853 ATCC (*P. aeruginosa*) reference strain. The in vitro antibacterial activity of the 7CrHAp and 7CrHAp-CH coatings was further evaluated against *Pseudomonas aeruginosa* 27853 ATCC (*P. aeruginosa*), *Escherichia coli* ATCC 25922 (*E. coli*) and *Staphylococcus aureus* ATCC 25923 (*S. aureus*) reference strains. In addition, atomic force microscopy (AFM) analysis was also used to investigate the ability of *P. aeruginosa*, *E. coli* and *S. aureus* cells to adhere and to develop colonies on the surfaces of the 7CrHAp and 7CrHAp-CH coatings. The results from the biological assays indicate that both coatings exhibit promising antibacterial properties, highlighting their potential for being used in biomedical applications, particularly in the development of novel antimicrobial devices.

## 1. Introduction

Repairing and regenerating hard tissues like bone remains a significant biomedical challenge today, as it requires materials that are highly biocompatible, osteoconductive, and ideally possess strong antimicrobial properties [1,2,3]. Bone tissue consists of 69% calcium phosphate (mainly hydroxyapatite), 21% collagen, 9% water, and 1% other components, giving it a composite structure of bioceramics and polymers [3]. Artificial bone biomaterials now include bioinert (e.g., alumina, zirconia), resorbable (e.g., tricalcium phosphate), and bioactive (e.g., hydroxyapatite) ceramics [3]. Hydroxyapatite (HAp, Ca_10_(PO_4_)_6_(OH)_2_), structurally similar to natural bone mineral, is widely used in orthopedics, dentistry, and drug delivery [1,2,3].

Natural polymers like chitosan and calcium phosphates (especially HAp and tricalcium phosphate) are commonly used in bone replacement, prostheses, coatings, and scaffolds [3,4,5,6,7,8]. HAp is the leading biomaterial for hard tissue regeneration due to its biocompatibility, accessibility, and abundance in mammalian tissues [4,5,6,7,8]. Chitin, the second most abundant polysaccharide, is a key structural component in many organisms [3]. Its derivative, chitosan, is a versatile, biocompatible biomaterial with biostimulatory, antibacterial, and anionic binding properties, making it useful in wound healing, tissue regeneration, pharmaceuticals, and other industries [9,10,11].

HAp’s crystal structure allows Ca^2+^ substitution with metal ions (e.g., Ag^+^, Fe^3+^, Zn^2+^), enhancing biocompatibility, osteoconductivity, antimicrobial activity, and mechanical strength [12,13,14,15,16]. Optimizing dopant levels is crucial to avoid cytotoxicity [12,13,14,15,16]. Chromium (Cr^3+^), essential for metabolism and collagen stability, is less toxic than Cr(VI), which poses significant health risks [17,18]. Predoi et al. [19] developed 20Cr-doped HAp thin films via spin coating, showing strong antifungal activity against *Candida albicans* and potential for biomedical coatings [19].

Ciobanu et al. [20] demonstrated that CrHAp and amoxicillin-loaded CrHAp coatings (produced by dip coating) exhibit high purity, biocompatibility, and antibacterial activity, especially against *Pseudomonas aeruginosa*, making them suitable for bone tissue engineering and implant coatings [20]. In another study, Ciobanu et al. [21] deposited CS–HAp composite layers using RF magnetron sputtering, which inhibited *Candida albicans* biofilm formation, highlighting their potential for antifungal coatings [21]. Thus, chromium-doped HAp/chitosan composites address the need for functional, safe biomaterials that prevent infections and promote tissue regeneration, offering significant promise for biomedical applications.

Here, we report the development of both chromium-doped hydroxyapatite (7CrHAp) and chromium-doped hydroxyapatite in chitosan matrix (7CrHAp-CH) composite layers by dip-coating technique. More than that, the new developed coatings were systematically investigated from physico-chemical and antimicrobial point of view. Data about the stability of the gels were achieved with the aid of the ultrasound (US) studies. The developed coatings were subjected to a comprehensive physico-chemical characterization—employing techniques such as XPS, FTIR, XRD, SEM, EDS and AFM—to evaluate their structural properties, chemical composition and surface morphology. In addition, their antimicrobial activity against the *Pseudomonas aeruginosa* ATCC 27853 reference strain was assessed through in vitro assays.

## 2. Materials and Methods

### 2.1. Materials

Chrome doped hydroxyapatite (7CrHAp, x_Cr_ = 0.07; [Ca + Cr]/P = 1.67) and chrome doped hydroxyapatite/chitosan (7CrHAp-CH) were obtained via a modified sol–gel route. The precursors used included: chromium nitrate nonahydrate (Cr(NO_3_)_3_·9H_2_O, 99.99%, Alfa Aesar, Karlsruhe, Germany), ammonium hydrogen phosphate ((NH_4_)_2_HPO_4_, 99.99%, Alfa Aesar, Karlsruhe, Germany), calcium nitrate tetrahydrate (Ca(NO_3_)_2_·4H_2_O, Sigma-Aldrich, St. Louis, MO, USA), chitosan (C_6_H_11_NO_4_, Sigma Aldrich, St. Louis, MO, USA), and ethanol. Dip coating method was employed for the deposition of the resulting 7CrHAp and 7CrHAp-CH sample on the silicon (Si) substrates.

#### Fabrication of Chrome Doped Hydroxyapatite and Chrome Doped Hydroxyapatite/Chitosan Samples

The detailed procedure used for the development of 7CrHAp and 7CrHAp-CH was described in detail in our recent paper [20,22,23]. Firstly, in the case of 7CrHAp sample, ammonium hydrogen phosphate was dissolved in 300 mL of ethanol and stirred (with 350 rpm) at 40 °C for 2 h. Thus, an 0.5 mol/L solution containing ammonium hydrogen phosphate was obtained. Then, separately, calcium nitrate and chromium nitrate were dissolved in 300 mL ethanol and stirred under similar conditions. Consequently, a 1.67 mol/L solution was obtained solution. The phosphate solution was slowly dropped into the nitrate solution under continuous stirring. The mixture was stirred at 80 °C for 12 h, maintaining the pH at 10. Afterward, the gel was washed five times with deionized water and ethanol, then redispersed in ethanol and stirred for an additional 12 h. On the other hand, a similar fabrication process was involved for the development of chrome-doped hydroxyapatite/chitosan (7CrHAp-CH) samples. For this sample (7CrHAp-CH), in the synthesis process the chitosan (6 g) was added into the calcium and chromium nitrate solution (300 mL, 1.67 mol/L) in order to obtain a solution with a 2% concentration of chitosan. The 7CrHAp and 7CrHAp-CH gels were employed for the deposition onto silicon substrates via the dip-coating technique, as previously presented [20,22,23]. Four consecutive layers of 7CrHAp and 7CrHAp-CH were deposited onto ultrasonically cleaned Si disks (which were rinsed with acetone and distilled water prior to deposition process). Each dip-coating cycle involved the immersion of the Si in the gels for several minutes (~5 min) and pulled out with a lifting speed of 7 μm/min using a homemade device. Each immersion was followed by thermal treatment at 80 °C for 4 h in air. After the final layer deposition, the coated Si substrates were annealed at 80 °C for 72 h [20,22,23] in air.

### 2.2. Methods

In order to obtain homogeneous and uniform layers we need stable suspensions. The stability of 7CrHAp and 7CrHAp-CH and Chitosan suspensions were evaluated using non-destructive ultrasound (US) measurements. A volume of 100 mL from each suspension (7CrHAp, 7CrHAp-CH and Chitosan) was stirred at room temperature for 15 min at 900 rpm to ensure optimal particle homogeneity. The prepared suspension was then transferred into a transparent cubic container equipped with two coaxial ultrasonic transducers, spaced 16 mm apart at the container’s mid-height. Immediately after stirring ceased, data acquisition commenced, with 1000 ultrasonic signals recorded at 5 s intervals using a digital oscilloscope for numerical analysis [20]. To reduce experimental noise, each recorded signal represents the average of 32 individual measurements. The stability of 7CrHAp and 7CrHAp-CH suspensions were evaluated using non-destructive ultrasound (US) measurements. Herein the bidistilled water was used as the reference medium. Each suspension (100 mL) was stirred at 900 rpm for 15 min at room temperature to ensure uniform mixing. The suspensions were then placed in a transparent cubic container fitted with two coaxial ultrasonic transducers, spaced 16 mm apart at the container’s mid-height. Finally, 1000 ultrasonic signals were recorded for analysis [20].

X-ray photoelectron spectroscopy (XPS) analysis was performed with the aid of a SPECS spectrometer equipped with a PHOIBOS 150 analyzer. Also, a Specs XR-50M RX source with a non-monochromatic magnesium anode (Ex = 1253.6 eV), operating at 300 W, was used to perform the XPS measurements [20]. For this work the charge compensation was achieved using a Specs FG15/40 flood gun and the spectral acquisition were carried out with a pass energy of 20 eV for high-resolution scans and 50 eV for survey scans. All binding energy (BE) values reported in this study were calibrated using the C1s peak centered at 284.8 eV. The experimental data were analyzed with the aid of CasaXPS 2.3.14 software (using Shirley background type) [20].

The structure of 7CrHAp and 7CrHAp-CH samples was analyzed by performing Xray diffraction (XRD) studies using a Bruker D8 Advance diffractometer with CuKα radiation (λ = 1.5418 Å) and a LynxEye™ detector. XRD data were collected from 20° to 70° (2θ) with a 0.02° step, and a 5s acquisition time per step.

The lattice parameters were calculated using Equation (1):

(1) $$\frac{1}{d^{2}}=\frac{4}{3}\left[\frac{h^{2}+k^{2}+hk}{a^{2}}\right]+\frac{l^{2}}{c^{2}}$$

The value of unit cell volume was determined using Equation (2):

(2) $$V=\frac{3\sqrt{3}}{2}a^{2}c$$

Average crystallite sizes were calculated using Equation (3)

(3) $$D_{hkl}=\frac{K\lambda }{\beta_{hkl}cos\theta_{hkl}}$$

In Equations (1)–(3), D is the average crystallite size, K is the Scherrer constant (0.94), β is the full width at half maximum (FWHM), (h, k, l) are the Miller indices, a and c are the lattice parameters, d is the interplanar spacing for the (hkl) planes, θ is the Bragg diffraction angle, and λ is the wavelength of the monochromatic X-ray beam (1.54 Å) [20].

FTIR spectroscopy was used to analyze the vibrations of functional groups present in the 7CrHAp and 7CrHAp-CH samples. The FTIR studies were made using a PerkinElmer spectrometer equipped with a Diamond/KRS-5 ATR accessory. Spectra were recorded from 450 to 2500 cm^−1^ with a 4 cm^−1^ resolution. FTIR second derivative (450–2500 cm^−1^) and FTIR deconvoluted spectra (900–1200 cm^−1^ and 1400–1700 cm^−1^) were also obtained following the procedures previously reported [24].

Scanning electron microscopy (SEM) and energy dispersive X-ray spectroscopy (EDS) analyses of the 7CrHAp and 7CrHAp-CH samples were carried out using a Hitachi S-4500 microscope (Hitachi, Tokyo, Japan) equipped with an EDAX system.

Surface topography of the 7CrHAp and 7CrHAp-CH samples was characterized by atomic force microscopy (AFM) using a NT-MDT NTEGRA Probe Nano Laboratory instrument (NT-MDT, Moscow, Russia). The AFM measurements were conducted in semi-contact mode at room temperature. AFM surface scans were performed over an area of 4.5 × 4.5 µm^2^. The root mean square roughness (R_RMS_) parameter was also determined. AFM image analysis and data processing were performed using Gwyddion software (version 2.59; Department of Nanometrology, Czech Metrology Institute, Brno, Czech Republic) [25].

Complementary data about the surface morphology of the 7CrHAp and 7CrHAp-CH samples, were obtained with the aid of metallographic microscopy (MM) analyses. The samples were examined using a 20× magnification objective on an inverted trinocular metallographic microscope (OX.2153-PLM, Euromex, Arnhem, The Netherlands). The acquired SEM, AFM and MM micrographs were processed and analyzed using ImageJ software (version 1.51j8), which was also employed to obtain three-dimensional surface profiles based on the metallographic images [26].

### 2.3. In Vitro Antibacterial Activity Assay

The antibacterial activity of the 7CrHAp and 7CrHAp-CH coatings was evaluated in vitro using the common reference strains, *Pseudomonas aeruginosa* 27853 ATCC, *Escherichia coli* ATCC 25922 and *Staphylococcus aureus* ATCC 25923. The experiments were performed according to a modified version of the protocol described by Ciobanu et al. [21], in accordance with the ISO 22196 standard method [27], which measures antibacterial performance by direct contact between bacterial suspensions and the coated surfaces. A standardized bacterial suspension having an initial concentration of 5 × 10^6^ CFU/mL was prepared and applied (100 μL per sample) to the surface of each coating to ensure an even coverage. The samples were incubated at 37 °C for 24, 48, and 72 h to assess the effect of the incubation time on the antibacterial performance of the tested samples. The bacterial viability was determined for each incubation time, and the results were expressed as log CFU/mL versus time. A positive control (C+), consisting of the bacterial suspension incubated without any coating, was used to establish the baseline for the bacterial growth. All the experiments were performed in triplicate, and the results were reported as mean values ± standard deviation (mean ± SD). Furthermore, atomic force microscopy (AFM) was also used to qualitatively assess the bacterial adhesion and colonization on the coating’s surfaces. For this purpose, after incubation, the samples were gently washed with sterile saline, fixed with cold methanol, and prepared for investigation. The AFM analyses were conducted in ambient conditions and the data was recorded in the noncontact mode. The scans were performed over 10 × 10 μm^2^ surface areas.

### 2.4. In Vitro Biological Evaluation

The cytotoxicity of the 7CrHAp and 7CrHAp-CH coatings was evaluated using human osteosarcoma MG63 cells (ATCC CRL-1427) [28], using a protocol previously described in detail by Iconaru et al. [29]. For this experiment, the MG63 cells were maintained in Dulbecco’s Modified Eagle Medium (DMEM) that was supplemented with fetal bovine serum (FBS) under standard culture conditions (37 °C, humidified atmosphere containing 5% CO_2_). The cells were then seeded at a density of 1 × 10^5^ cells per well and exposed to the coatings for 24 and 48 h. The cell viability was quantified using the MTT reduction assay, which measures the mitochondrial metabolic activity. After incubation with the coatings, the MTT reagent was added, and the resulting formazan crystals were solubilized before recording the absorbance at 595 nm using a TECAN spectrophotometer (TECAN, Männedorf, Switzerland). The percentage of viable MG63 cells was determined reported to a control sample, which was considered to have 100% viability. The percentage of viable cells was calculated relative to the untreated control group, which was set at 100%. After 48 h, the coatings were gently rinsed with sterile saline solution, fixed with cold methanol, and prepared for microscopic examination in order to investigate the cells adherence and development on the coatings surface. The morphological evaluation of the MG63 cells was carried out by SEM and MM. Digital micrographs were further analyzed with ImageJ software (version 1.51j8) to quantify changes in cell morphology and coverage [26].

## 3. Results

**Figure 1:**
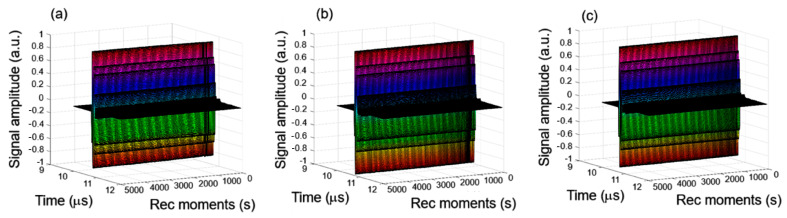
Evolution of the recorded ultrasonic signals as a function of time over 5000 s for the 7CrHAp (**a**), 7CrHAp-CH (**b**), and CH (**c**) suspensions, presented sequentially from right to left.

Figure 1 represents the superimposed set of 1000 recorded ultrasonic signals for 7CrHAp (Figure 1a), 7CrHAp-CH (Figure 1b), and Chitosan (Figure 1c). The signals, corresponding to 5000 s of process evolution, are displayed in a waterfall-style plot from right to left. In this representation, the sedimentation process appears to occur extremely slowly and remains imperceptible.

**Figure 2:**
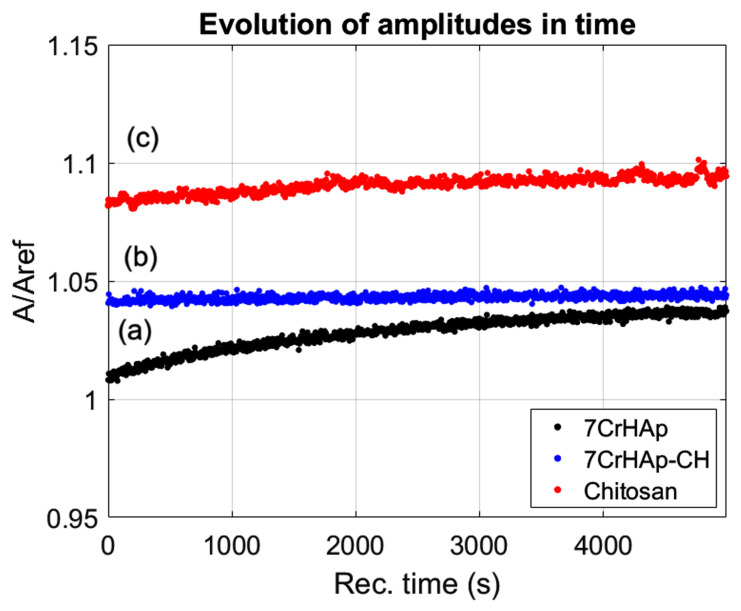
Evolution of the recorded signal amplitudes over time for the 7CrHAp (**a**), 7CrHAp-CH (**b**), and Chitosan (**c**) suspensions, throughout the experimental duration.

While, in Figure 2 the time evolution of the ultrasonic amplitudes for 7CrHAp, 7CrHAp-CH, and Chitosan suspensions are presented. For all three suspensions, the amplitude exhibited a gradual increase over time, indicating a good stability and minimal sedimentation within the observation window. For 7CrHAp suspension (black dots), the amplitude increases steadily with no sharp or abrupt signal variations, thus showing a stable system. For the second suspension, 7CrHAp-CH (blue dots), no rapid signal fluctuations were detected, indicating that the overall system remained stable. For the Chitosan suspension (red dots), the signal amplitude remained constant throughout the entire duration, reflecting a highly stable suspension.

**Figure 3:**
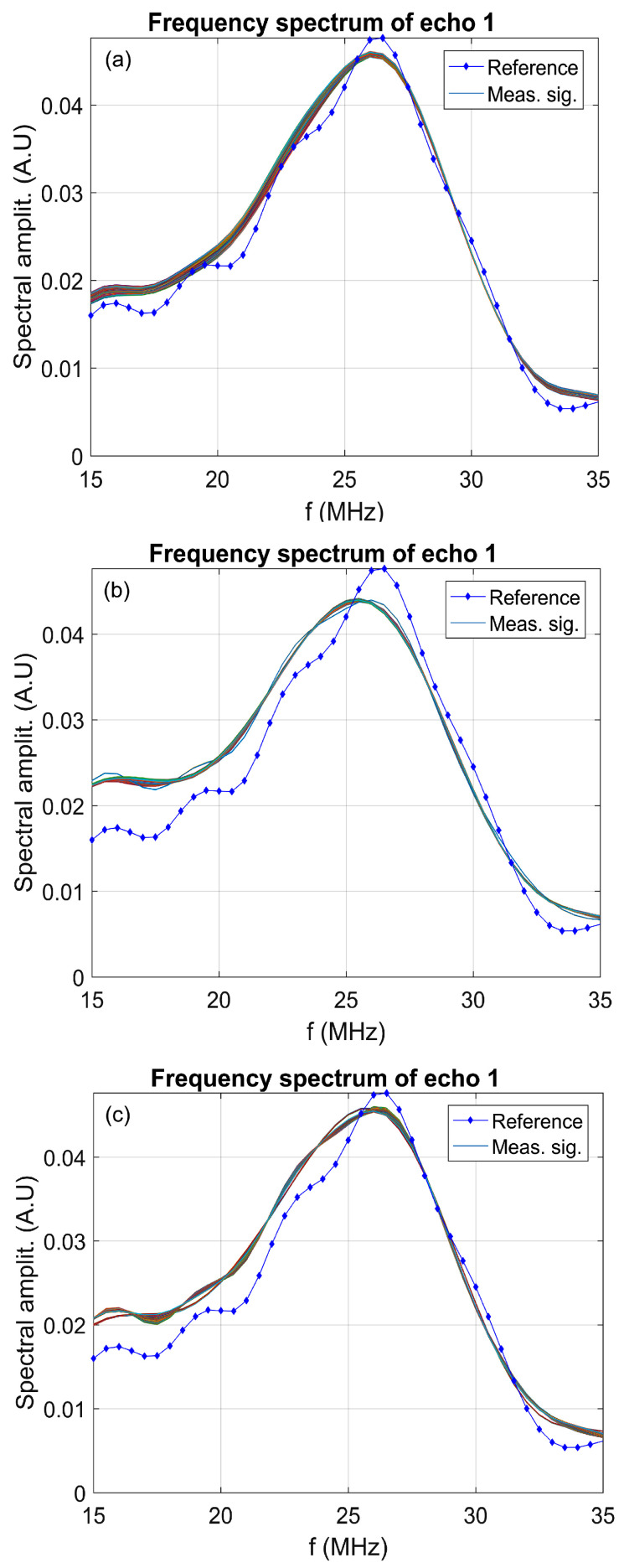
Spectral amplitudes of ultrasonic signals recorded for 7CrHAp (**a**), 7CrHAp-CH (**b**), and CH (**c**) suspensions.

Figure 3 presents the evolution of the frequency spectrum corresponding to the 1000 recorded ultrasonic signals. For reference, the spectrum of double-distilled water is included as a dotted blue line for comparison. As observed, all samples exhibit a primary amplitude peak around 26 MHz, which corresponds to the central operating frequency of the ultrasonic transducers. For 7CrHAp (Figure 3a), the spectral curve is consistently above the reference in the range of approximately 15–26 MHz, which indicates that 7CrHAp exhibits a lower attenuation than water in this frequency range, and while it does not overlap perfectly with the reference line, the similar spectral shape and slightly consistent higher amplitudes suggest a stable suspension with a slightly enhanced transmission, due to the elasticity of the nanoparticles. In contrast, 7CrHAp-CH (Figure 3b) shows a spectral amplitude that is slightly above the reference and deviates more noticeably at lower frequencies. This behavior may be attributed to small variations in particle distribution, such as early-stage sedimentation or mild clustering. Despite this, 7CrHAp-CH still demonstrates good spectral stability and retains a shape close to the reference, confirming that the suspension remains relatively homogeneous over the measurement period; thus, the suspension is also highly stable. For Chitosan (Figure 3c), the spectral profile shows an almost perfect overlap with the reference signal across the entire frequency range, with the amplitudes matching very closely, indicating that Chitosan mimics the acoustic behavior of water with remarkable precision, pointing to exceptional suspension stability, with minimal attenuation and negligible scattering or absorption by particles. To conclude, all three suspensions exhibit good stability, as reflected by their smooth spectral profiles and minimal deviation from the reference liquid, with Chitosan standing out as the most acoustically stable and homogeneous after the sudden precipitation, followed closely by 7CrHAp-CH, which shows slightly enhanced characteristics, while 7CrHAp remains stable but presents the most variation.

**Figure 4:**
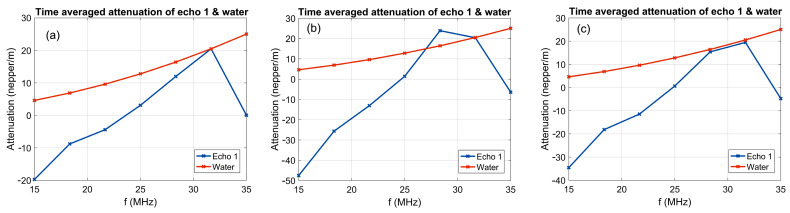
Time averaged attenuation for the investigated frequency range for 7CrHAp (**a**), 7CrHAp-CH (**b**), and CH (**c**) suspensions.

Figure 4 shows the average amplitudes in the frequency spectrum corresponding to the 1000 recorded ultrasonic signals, where the natural logarithm of the amplitude ratios relative to the reference liquid was calculated and displayed. The first suspension, 7CrHAp (Figure 4a), exhibits lower attenuations compared to the reference liquid in the frequency range of the ultrasonic transducer, with a pronounced peak at 32 MHz, where there is a good superposition of amplitudes with the reference liquid, followed by a sharp decline. The 7CrHAp-CH suspension (Figure 4b) also exhibits a lower attenuation in the lower frequency range compared to the reference liquid, however the signal is characterized by a smooth incline, until at a frequency of 28 MHz, the attenuation of the sample exceeds that of the reference liquid by 7–8 neper/m. The Chitosan suspension (Figure 4c) exhibits lower attenuation than water across most of the investigated frequency range, with the attenuation curve closing on that of the reference liquid around 28–32 MHz, but without intersecting it. This close proximity suggests that the suspension has acoustic properties comparable to water at that frequency range, showing that the Chitosan suspension is highly stable.

In order to assess the level of stability of the samples, a quantitative stability parameter $S=\bar{\frac{dA}{Adt}}$ was calculated; the values obtained are as follows: 2.59·10^−6^ s^−1^ for sample 7CrHAp, 8.64·10^−7^ s^−1^ for sample 7CrHAp-CH and 3.14·10^−7^ s^−1^ for sample CH. These results indicate that all samples are stable, with Chitosan being extremely stable, followed by 7CrHAp-CH, which is very stable, and 7CrHAp which is stable.

**Figure 5:**
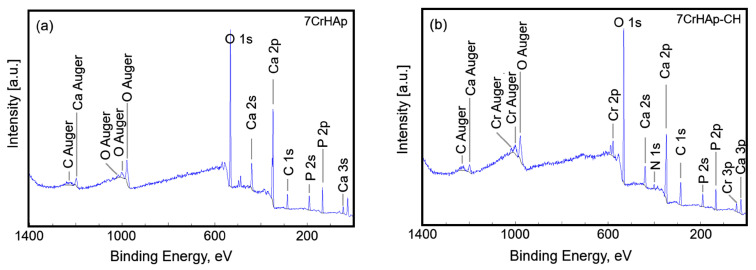
Survey scan XPS spectra of the 7CrHAp (**a**) and 7CrHAp-CH (**b**) materials registered at room temperature.

This study aimed to determine the surface composition of the 7CrHAp and 7CrHAp-CH materials by X-ray photoelectron spectroscopy (XPS). The XPS analysis focuses on evaluating the accuracy of XPS peak decomposition and component assignment for 7CrHAp and 7CrHAp-CH materials. Figure 5 presents the general XPS spectra for the 7CrHAp and 7CrHAp-CH samples. The observed peaks correspond to the elemental components of each material. Table 1 presents the surface atomic composition (atomic %) of the 7CrHAp and 7CrHAp-CH samples.

**Figure 6:**
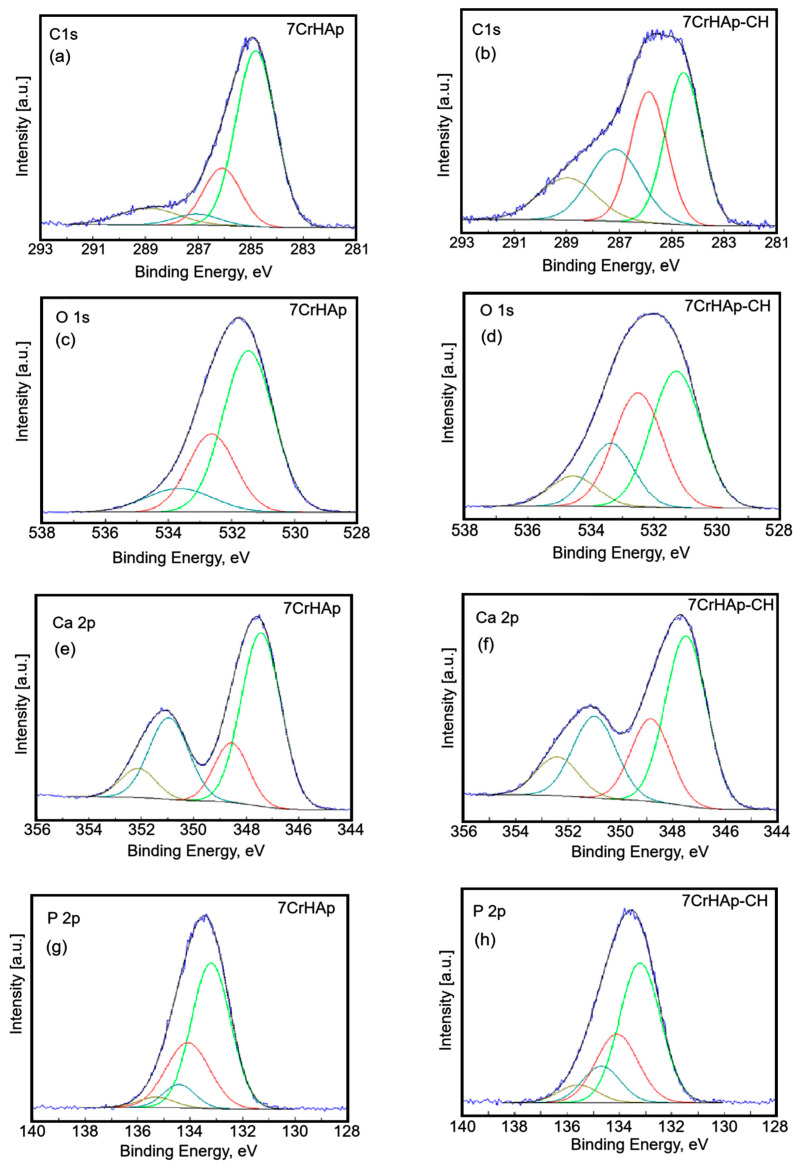
XPS high-energy resolution spectra of C 1s (**a**,**b**), O 1s (**c**,**d**), Ca 2p (**e**,**f**) and P 2p (**g**,**h**) peaks pour 7CrHAp and 7CrHAp-CH samples.

**Figure 7:**
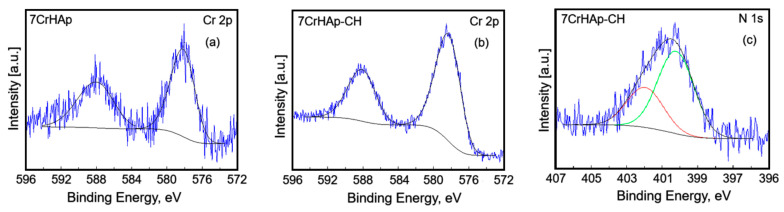
XPS high-energy resolution spectra of Cr 2p (**a**,**b**) and N 1s (**c**) peaks for 7CrHAp and 7CrHAp-CH samples.

The C 1s, O 1s, Ca 2p, P 2p, Cr 2p and N 1s peaks were decomposed using a least square fitting procedure with a Gaussian-Lorentzian product function. Figure 6 and Figure 7 presents the high-resolution XPS spectra for the elements mentioned above for both samples, highlighting the detailed chemical states of C 1s, O 1s, Ca 2p, P 2p, Cr 2p, and N 1s.

The C 1s peak of 7CrHAp and 7CrHAp-CH materials was decomposed in four components (Figure 6a,b). The first component was observed at 284.8 eV for both samples. The second component was identified at 286.08 (7CrHAp) and 286.13 eV (7CrHAp-CH). The third component was observed at 287.06 (7CrHAp) and 287.41 eV (7CrHAp-CH) while the fourth component was located at 288.88 (7CrHAp) and 289.23 eV (7CrHAp-CH). The first component is typical of carbon bonded only to carbon and hydrogen [C ― (C, H)]. The second component can be attributed to carbon forming a single bond with oxygen or nitrogen [C― (O, N)]. The third component is typical of O ― C ― O and N ― C = O bonds [30,31,32,33]. The fourth component is attributed to contaminants containing ―COOR groups.

Figure 6 shows the high-resolution spectra of C 1s, O 1s, Ca 2p and P 2p of the 7CrHAp and 7CrHap-CH samples.

The O 1s peak of 7CrHAp and 7CrHAp-CH materials (Figure 6c,d) was decomposed in three components. The firs component was found at 531.47 (7CrHAp) and 531.29 eV (7CrHAp-CH). The second component was identified at 532.63 (7CrHAp) and 532.50 eV (7CrHAp-CH). The third component was observed ad 533.66 (7CrHAp) and 534.56 eV (7CrHAp-CH). The firs component can be allocated to oxygen in hydroxyapatite and PO_4_^3−^ groups of HAp including C = O double bonds. The second component is assigned to the C ― O and C ― OH bound and O ― C ― O chemical bindings from the CH [31]. The third component can be attributed to traces of adsorbed water and possible contaminants with C ― O single bonds.

The high-resolution XPS spectra of Ca 2p (Figure 6e,f) reveal four distinct components. The primary doublet, corresponding to Ca 2p_3_/_2_ and Ca 2p_1_/_2_, appears at binding energies of 347.43 eV and 350.95 eV for the 7CrHAp sample, and at 347.49 eV and 350.99 eV for the 7CrHAp-CH sample. These peaks are separated by approximately 3.6 eV in both cases, with an area ratio of 2:1, which is characteristic of calcium in hydroxyapatite. Additionally, a secondary doublet is observed at 348.56 eV and 352.12 eV for 7CrHAp, and at 348.84 eV and 352.41 eV for 7CrHAp-CH. This higher-energy doublet suggests the presence of calcium in a more oxidized state.

The high-resolution XPS spectrum of P 2p (Figure 6g,h) exhibited four distinct components. The primary doublet, corresponding to P2p_3/2_ si P2p_1/2_, appears at binding energies of 133.22 and 134.10 eV for the 7CrHAp sample, and at 132.97 eV and 133.86 eV for the 7CrHAp-CH sample. These peaks are separated by approximately 0.9 eV for both samples, with an area ratio of 2:1, which is characteristic of hydroxyapatite (― PO_4_), in accordance with anterior studies [33]. Additionally, a secondary doublet is observed at 134.42 eV and 135.50 eV for 7CrHAp, and at 134.70 eV and 135.58 eV for 7CrHAp-CH. This higher-energy doublet suggests the presence of phosphorus in a more oxidized state.

The high-resolution XPS spectrum of Cr 2p (Figure 7a,b) revealed two distinct components. The first peak was observed at BE of 578.18 (7CrHAp) and 578.30 eV (7CrHAp-CH). This peak corresponds to the Cr 2p_3/2_ orbital. In the context of chromium-doped hydroxyapatite, this peak is often associated with the presence of Cr(III) (chromium in the +3-oxidation state). The second peak were identified at BE of 588.06 (7CrHAp) and 588.13 eV (7CrHAp-CH). This peak corresponds to the Cr 2p_1/2_ orbital. Similar to the Cr 2p_3/2_ peak, this is also indicative of Cr(III). The separation between the Cr 2p_3/2_ and Cr 2p_1/2_ peaks is typically around 9–10 eV, which is consistent with the obtained values. In summary, these peaks suggest the presence of chromium in the +3-oxidation state within the hydroxyapatite matrix. These results were in agreement with previous studies [34,35].

The peaks at 400.23 eV and 401.96 eV in the high-resolution XPS spectrum of the N1 core level for chromium-doped hydroxyapatite (7CrHAp-CH) in a chitosan matrix can be attributed to specific nitrogen environments influenced by the chemical structure and interactions within the composite (Figure 7c). The peaks at 400.23 eV and 401.96 eV in the high-resolution XPS spectrum of the N1 core level for chromium-doped hydroxyapatite in a chitosan (7CrHAp-CH) matrix can be attributed to specific nitrogen environments influenced by the chemical structure and interactions within the composite.

The N 1s peak at ~400.23 eV in the XPS spectrum of 7CrHAp-CH is typically attributed to nitrogen in amine or amide groups, specifically protonated amines (― NH_3_^+^) or neutral amines (― NH_2_) that are part of the chitosan polymer structure [36]. In the situation of 7CrHAp-CH, the peak at 400.23 eV most likely arises from chitosan’s primary amine groups interacting with the hydroxyapatite surface and/or chromium ions. These interactions, along with the conditions during synthesis, may lead to protonation of the amine groups, contributing to the observed binding energy. It may also indicate nitrogen atoms involved in coordination with chromium ions, forming Cr ― N bonds. In chitosan, depending on the pH and the chemical environment, some of the amine groups can become protonated, leading to a slight shift in the binding energy. These interpretations are consistent with typical XPS data for chitosan and its derivatives [36,37].

As can be seen in the XPS spectra analyzed in this study, the C 1s peak has a higher intensity in the XPS spectrum of 7CrHAp-CH. The higher intensity of the C 1s peak in the XPS spectrum of CrHAp-Chitosan compared to CrHAp is primarily due to the presence of chitosan in the composite. Chitosan is a polysaccharide derived from chitin and contains a high proportion of carbon atoms in its molecular structure. When chitosan is added to CrHAp, it significantly increases the total carbon content of the sample. The C 1s signal observed in the XPS spectrum of CrHAp is attributed to surface contamination (e.g., adsorbed CO_2_ or hydrocarbons from the environment). The diminished C 1s peak at ~287 eV in 7CrHAp compared to 7CrHAp-Chitosan (7CrHAp-CH) is a telling indicator of the chemical environment and surface composition differences between the two materials. The 7CrHAp sample is an inorganic material, primarily composed of calcium, phosphate, and chromium ions, which makes the peak at 287 eV, corresponding to O ― C ― O and N ― C = O bonds, smaller. The surface of 7CrHAp is dominated by PO_4_^3−^, OH^−^, and possibly CO_3_^2−^ substitutions, but not by amide or ester groups [38,39,40]. Any carbon signal is mostly from adventitious contamination, which typically appears around 284.8 eV (C ― C/C ― H). According to the results of the present study, we can say that the diminished 287 eV peak in 7CrHAp and its enhancement in 7CrHAp-CH confirms the successful incorporation of chitosan and highlights the presence of amide and ester-like functionalities introduced by the polymer. Similarly, for the high-resolution O 1s XPS spectra, there is an additional peak between 534 and 532 eV (Figure 6d) for 7CrHAp-CH4, which is not observed in 7CrHAp.

The additional peak between 534 and 532 eV in the O 1s XPS spectrum of 7CrHAp-CH, which is not observed in 7CrHAp, can be attributed to the fact that chitosan contains several oxygen-containing functional groups such as oxygen ca hydroxyl (― OH), ether (C ― O ― C), amide (O = C ― N ―), adsorbed water (H_2_O) that contribute to higher binding energy components in the O 1s spectrum, typically appearing as a shoulder or distinct peak in the 532–534 eV range. Therefore, the emergence of a new O 1s peak in 7CrHAp-CH is likely attributed to oxygen-containing functional groups introduced by chitosan, along with surface adsorbates such as C ― O, C = O/COO^−^, and H_2_O. These species typically appear in the 532–534 eV range and are not present in the unmodified 7CrHAp sample [41,42].

The successful incorporation of chitosan into the 7CrHAp-CH sample was also validated by the presence of N 1s peak at ~400.23 eV in the XPS spectrum of 7CrHAp-CH. Moreover, this peak suggests possible interactions between chitosan and Cr-doped hydroxyapatite, which may contribute to the enhancement of antimicrobial properties.

**Figure 8:**
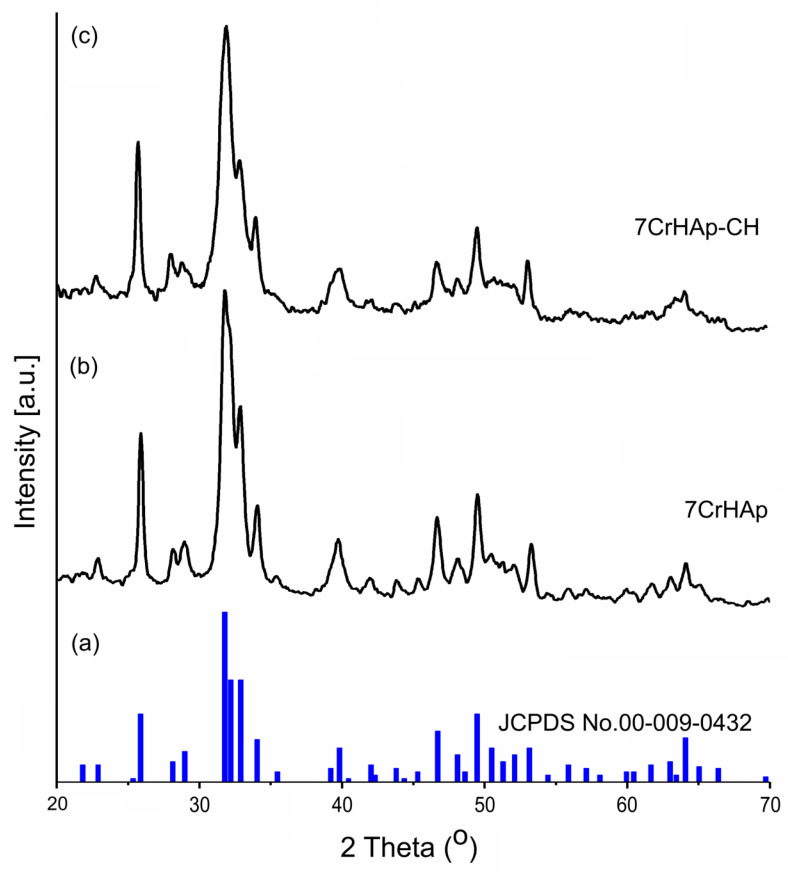
X-ray diffraction spectra of the 7CrHAp (**b**) and 7CrHAp-CH (**c**) sample. Characteristic diffraction pattern of pure hexagonal hydroxyapatite, JCPDS No. 00-009-0432 (**a**).

In this study, XRD analysis was used to identify the mineral phase and to confirm the change in the crystallinity of the 7CrHAp and 7CrHAp-CH coatings. Figure 8 illustrates the XRD spectra of the 7CrHAp samples (Figure 8b) and 7CrHAp-CH (Figure 8c) as well as the characteristic diffraction pattern of pure hexagonal hydroxyapatite, JCPDS No. 00-009-0432, (Figure 8a). A broadening of the diffraction peaks of the 7CrHAp-CH sample (Figure 8c) is observed. This broadening can be attributed to both the decrease in crystallite size and the decrease in crystallinity of the sample due to the presence of CH. In agreement with previous studies [43] the slight decrease in crystallinity in the presence of CH could be explained by weaker hydrogen bonding interactions between chains in the 7CrHAp-CH sample.

As can be seen in Figure 8 both the 7CrHAp and 7CrHAp-CH samples show specific peaks of pure hexagonal HAp in accordance with the diffraction data sheet JCPDS No. 00-009-0432. Thus, the X-ray diffraction (XRD) spectra reveal the presence of the main characteristic peaks of hexagonal HAp crystals associated with the reflections due to (002), (102), (210), (211), (300), (202), (310), (222), (213), (004). The XRD spectra of the two analyzed materials show obvious similarities in their characteristic peaks. However, a slight difference was observed in terms of peak broadening. Also, a slight decrease in the average crystallite size was observed in the case of the 7CrHAp-CH sample. The lattice parameters, unit cell volume and average crystallite size of the two analyzed materials are presented in Table 2.

**Figure 9:**
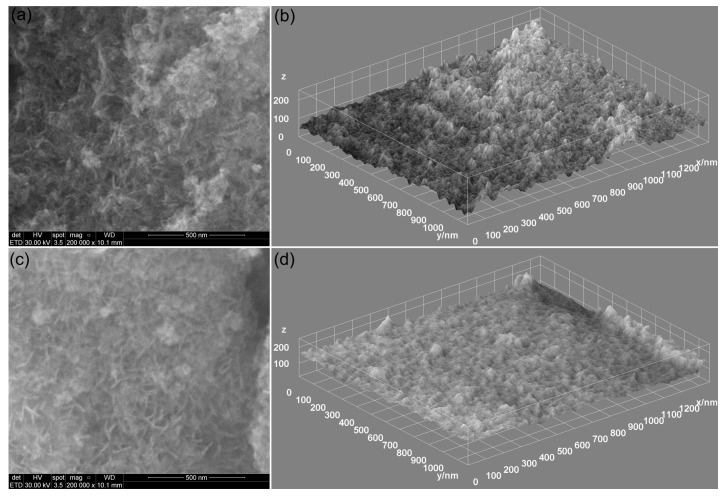
2D and 3D SEM micrographs obtained for the 7CrHAp (**a**,**b**) and 7CrHAp-CH (**c**,**d**).

In order to obtain preliminary data on the surface morphology of 7CrHAp and 7CrHAp-CH samples, scanning electron microscopy (SEM) analyses were performed. The obtained results, including both two-dimensional (2D) and three-dimensional (3D) SEM micrographs, are depicted in Figure 9. Accordingly, the SEM analysis of the 7CrHAp sample (Figure 9a,b) reveals the presence of a nanostructured and continuous surface, free of cracks and other observable surface defects. Furthermore, analysis of the 2D and 3D SEM images characteristic of the 7CrHAp-CH sample (Figure 9c,d) indicates that the incorporation of chitosan induces slight modifications in the surface morphology, while preserving a continuous, nanostructured and homogeneous surface structure.

**Figure 10:**
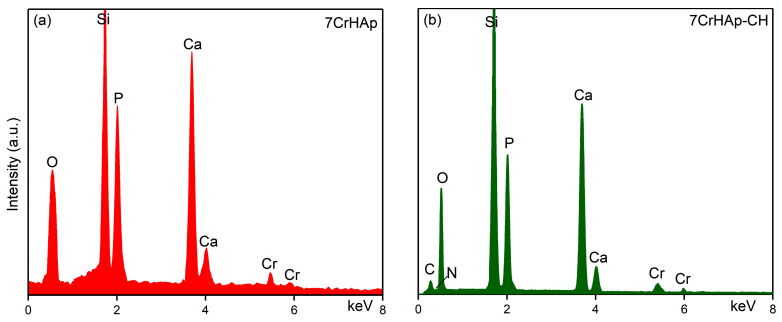
EDS spectra of 7CrHAp (**a**) and 7CrHAp-CH (**b**) samples.

The results of EDS analysis performed on the 7CrHAp and 7CrHAp-CH samples are presented in Figure 10. The results of EDS analysis identified the presence of calcium, chromium, oxygen and phosphorus in the 7CrHAp sample (Figure 10a). On the other hand, in the case of the 7CrHAp-CH sample the EDS analysis confirmed the presence of the main constituent elements of the chromium doped hydroxyapatite/chitosan (nitrogen, carbon, oxygen, calcium, chromium and phosphorous) in the analyzed sample (Figure 10b). In both EDS spectra, the presence of the Si line is detected, which appears due to the substrate on which the samples were deposited. Furthermore, the EDS results clearly highlight the absence of additional lines that could indicate the presence of impurities (Figure 10). The absence of carbon in the EDS spectrum of 7CrHAp is due to the fact that EDS detects X-rays emitted from deep within the sample (down to 1–2 microns) while XPS only probes the top of a material (~1–10 nm). Since the carbon present in 7CrHAp originates from surface contamination, its low-energy X-rays are readily absorbed and fail to reach the EDS detector [44,45,46,47].

Additional information regarding the quantitative chemical composition of both analyzed samples were obtained and by EDS studies. The results of the EDS semiquantitative studies are revealed in Table 3. Thus, could be noticed that for the sample 7CrHAp the value of the (Ca+Cr)/P ratio was equal with 1.72. Meanwhile, the value of (Ca+Cr)/P ratio obtained for 7CrHAp-CH sample was 1.68.

**Figure 11:**
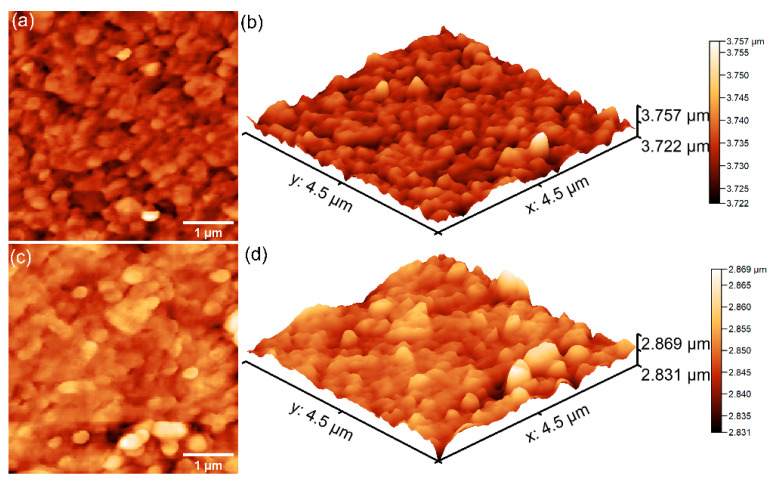
2D and 3D AFM topographies obtained for the 7CrHAp (**a**,**b**) and 7CrHAp-CH (**c**,**d**).

Further details concerning the surface topography of the 7CrHAp and 7CrHAp-CH samples were acquired through atomic force microscopy (AFM). The corresponding two- and three-dimensional AFM images are displayed in Figure 11.

AFM analysis revealed that the surface topographies are consistent with those revealed by the SEM studies, indicating the presence of continuous, homogeneous, and well-structured surfaces. More than that, both samples exhibited smooth coating surfaces, free of fissures and cracks. In addition, the AFM results indicated that the addition of chitosan induce changes in surface roughness as reflected by the root mean square (R_RMS_) values: 14.12 nm for 7CrHAp and 7.106 nm for 7CrHAp-CH sample. The polymer matrix led to the reduction of 7CrHAp particle agglomeration, leading to a more homogeneous dispersion in the 7CrHAp-CH sample. Furthermore, the granular texture in the case of the 7CrHAp-CH sample was slightly smoothed (due to the presence of chitosan) which led to a flatter surface [48]. These findings confirm that addition of chitosan influences the surface morphology and are in good agreement with the SEM observations.

**Figure 12:**
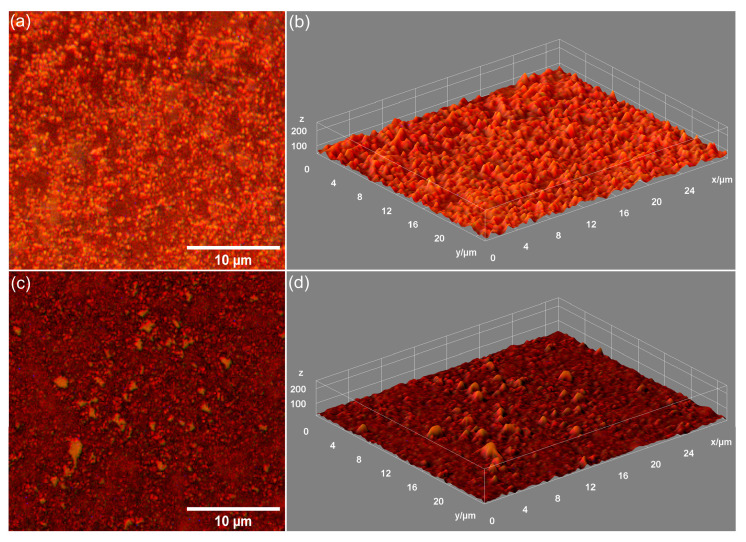
2D and 3D MM images obtained for the 7CrHAp (**a**,**b**) and 7CrHAp-CH (**c**,**d**).

Metallographic microscopy provided additional data about the surface morphology of the 7CrHAp and 7CrHAp-CH samples. The 2D metallographic images (recorded at 20× magnification) and their 3D representation are shown in Figure 12.

The obtained metallographic microscopy images indicate that the surface of both 7CrHAp and 7CrHAp-CH samples is well-structured, free of cracks or fissures. More than that, the 3D metallographic images confirm that these surfaces (of 7CrHAp and 7CrHAp-CH) display the typical features of a continuous and relatively uniform deposited layer. These findings are consistent with SEM and AFM results, which also demonstrate a defect-free, continuous, well-structured and uniform surface morphology of the studied samples. Our results, obtained using three complementary microscopy techniques, underline the fact that the dip-coating deposition method employed for the fabrication of the 7CrHAp and 7CrHAp-CH samples is effective in producing surfaces that are continuous, homogeneous, and free of major morphological defects.

**Figure 13:**
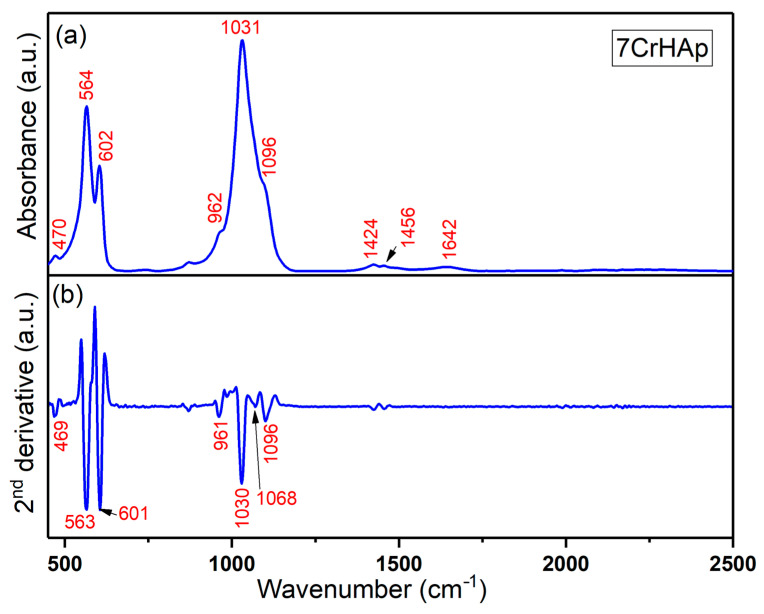
FTIR general spectra (**a**) and FTIR second derivative spectra (**b**) characteristic of 7CrHAp sample recorded between 450 and 2500 cm^−1^.

**Figure 14:**
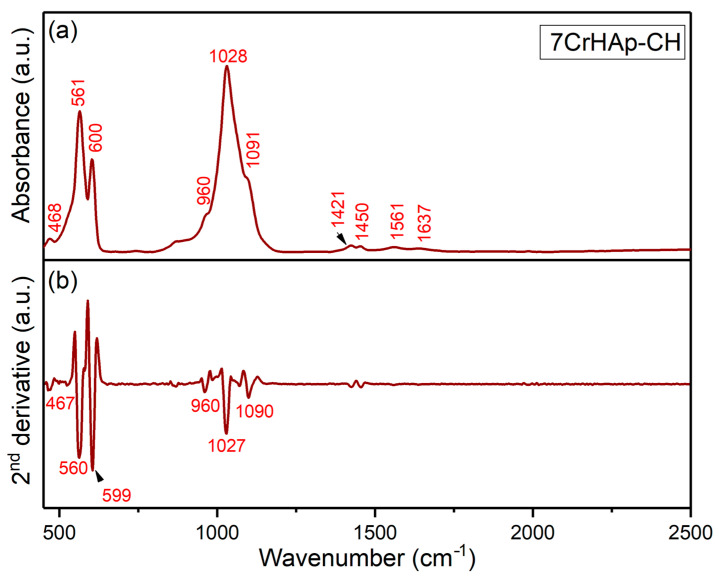
FTIR general spectra (**a**) and FTIR second derivative spectra (**b**) obtained for 7CrHAp-CH sample recorded between 450 and 2500 cm^−1^.

Both FTIR general spectra and FTIR second derivative spectra of 7CrHAp and 7CrHAp-CH samples are revealed in Figure 13 and Figure 14. For chromium doped hydroxyapatite (Figure 13), the absorption bands observed at around 470, 564, 602, 962, 1031, and 1096 cm^−1^ could be manly attributed to the PO_4_^3−^ stretching (P – O) and bending (O – P – O) vibrations (ν_2_, ν_3_, ν_4_, and ν_1_) [20,49]. The FTIR absorption bands centered at approximately 1424 cm^−1^ and 1456 cm^−1^ in chromium-doped hydroxyapatite are mainly assigned to the C – O asymmetric stretching and bending vibrational modes of carbonate (CO_3_^2−^) functional groups [20,49,50]. These bands indicate the presence of carbonate substitutions within the hydroxyapatite lattice, commonly classified as type A or type B substitutions, which result in partial replacement of phosphate or hydroxyl groups. The band observed near 1642 cm^−1^ is usually attributed to the bending vibrations of O – H groups from adsorbed or structural water within the sample [20,49,50]. For pure hydroxyapatite, the 1642 cm^−1^ feature is generally indicative of O – H stretching vibrations of adsorbed water [50].

The general FTIR spectra and second derivative spectra of the 7CrHAp-CH sample (Figure 14) reveal vibrational bands characteristic of phosphate groups. The symmetric stretching mode (ν_1_) is observed near 960 cm^−1^ [20,49,50,51], while the triple-degenerate asymmetric stretching vibrations (ν_3_) of the P – O bonds appear between 1000 and 1100 cm^−1^. Additionally, the double-degenerate bending mode (ν_2_, O – P – O) is typically detected within the 400–500 cm^−1^ range, and the triple-degenerate bending vibrations (ν_4_, O – P – O) are found in the 550–600 cm^−1^ spectral region [20,49,50,51].

Furthermore, the FTIR general spectra of the 7CrHAp-CH sample reveal that the addition of chitosan induces a shift in the positions of the main peaks (to lower wavelengths), accompanied by a slight broadening of these peaks. More than that, in the FTIR general spectra of 7CrHAp-CH the vibrational bands observed in the spectral region 1300–1800 cm^−1^ are predominantly associated with characteristic functional groups present in the chitosan structure [51]. Specifically, around ~1561 cm^−1^ is assigned to N – H bending vibrations (amide II) [51]. Additional bands in this region, such as those near 1420–1450 cm^−1^, are assigned to C – H bending [51]. These assignments are consistent with the FTIR spectra of chitosan previously reported in the literature [51], confirming the presence of the chitosan. In addition, in this spectral region, the absorption bands are overlapped with the spectral features attributable to the vibrations of the carbonate group. The observed vibrational features highlight the complex molecular environment of chitosan and chromium doped hydroxyapatite, reflecting the presence of characteristic functional groups from both components. These functional groups are essential for their chemical reactivity and potential applications in biomedical fields. Table 4 summarizes the main peaks observed in both FTIR spectra along with their corresponding assignments.

**Figure 15:**
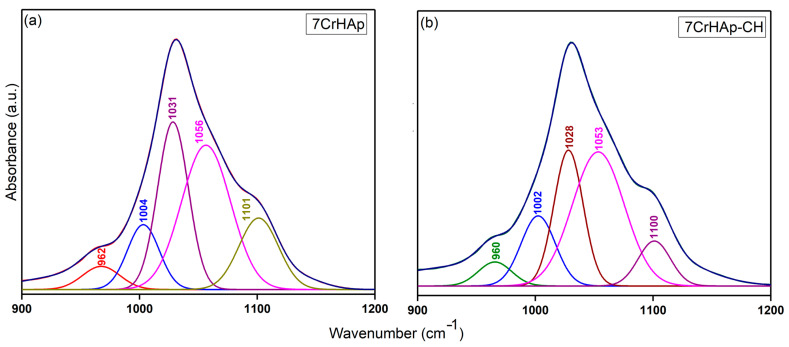
Deconvoluted FTIR spectra of 7CrHAp (**a**) and 7CrHAp-CH (**b**) samples in the 900–1200 cm^−1^ region, showing the phosphate vibrational bands (ν_1_ and ν_3_) typical of the HAp structure, along with potential overlapping signals from chitosan.

To more clearly highlight the presence of overlapped vibrational bands in the FTIR general spectra of the two samples, the obtained spectra were deconvoluted and analyzed in a spectral region that includes the maxima corresponding to both HAp and chitosan functional groups. Thus, Figure 15 shows the deconvoluted FTIR spectra of 7CrHAp and 7CrHAp-CH samples in the range of 900–1200 cm^−1^, characteristic of the ν_1_ and ν_3_ vibrational modes of the phosphate functional groups (P – O vibration) in the HAp structure. Thus, could be clearly observed that five components were used to obtain a good fit for the 7CrHAp and 7CrHAp-CH samples. In addition, the deconvoluted spectra of the 7CrHAp-CH sample exhibited a decrease in the intensity of the subbands within the 1000–1100 cm^−1^ spectral region, corresponding to the ν_3_ vibrational mode of phosphate in HAp, compared to the subbands observed in the FTIR deconvoluted spectra of 7CrHAp sample.

**Figure 16:**
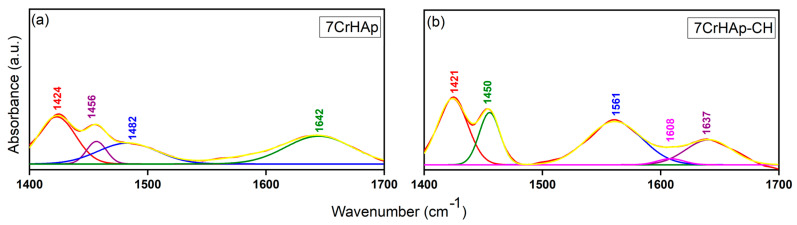
Deconvoluted FTIR spectra of 7CrHAp (**a**) and 7CrHAp-CH (**b**) samples in the 1400–1700 cm^−1^ region, highlighting the carbonate vibrational bands of HAp and additional contributions from chitosan-related functional groups (e.g., –NH_2_ and –OH).

More than that, in Figure 16 are depicted the deconvoluted FTIR spectra of 7CrHAp and 7CrHAp-CH samples obtained in the 1400–1700 cm^−1^ spectral range which is characteristic for the vibrational modes associated with carbonate functional groups (C – O) in the HAp structure, as well as vibrations originating from functional groups within the chitosan molecular structure. Accordingly, four individual subbands were necessary to achieve an optimal fit for the deconvoluted spectra of 7CrHAp samples in the 1400–1700 cm^−1^ region. Additionally, in the case of the 7CrHAp-CH sample, five subbands were required to achieve the best fit of the deconvoluted FTIR spectrum in the 1400–1700 cm^−1^ region. This increase in the number of components, compared to the 7CrHAp sample, reflects the contribution of additional vibrational modes introduced by chitosan, likely associated with its characteristic functional groups such as –NH_2_ and –OH, which overlap with or modify the existing carbonate-related absorptions within the HAp.

Notably, the incorporation of chitosan in the 7CrHAp-CH sample resulted in an overall increase in the intensity of the absorption bands within this spectral domain (1400–1700 cm^−1^). This enhancement could be an indicative of additional vibrational contributions from the functional groups present in the chitosan structure which overlap with or increase the characteristic carbonate-related vibrational modes (C – O) of the HAp. These results are consistent with previous findings [20,49,50,51], further supporting the spectral interpretations presented in this study and indicating the formation of the composite coatings and the complex interaction between HAp and chitosan [20,49,50,51].

**Figure 17:**
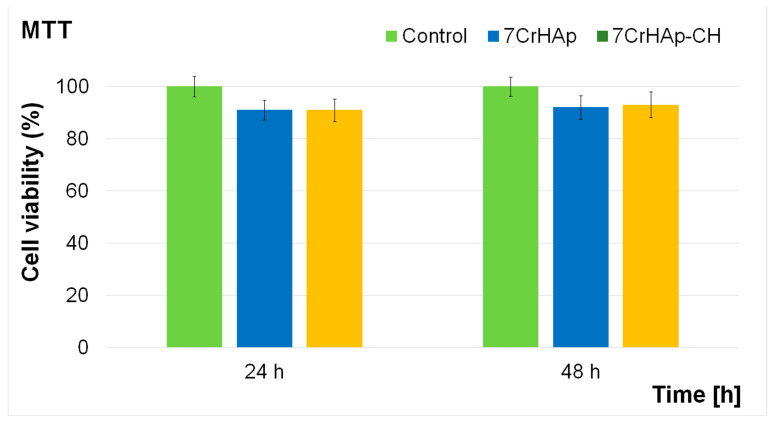
Cell viability of MG63 cells incubated with 7CrHAp and 7CrHAp-CH for 24 and 48h. The results are represented as mean ± standard deviation (SD) and are expressed as percentages of control (100% viability).

The cytotoxicity of 7CrHAp and 7CrHAp-CH coatings was assessed using the MTT colorimetric assay with the aid of MG63 cell line. The cell viability was determined after 24 and 48 h of the cells incubation with the coatings and expressed as mean ± standard deviation (SD) relative to the control (100% viability). The results obtained from the in vitro MTT assay are presented in Figure 17.

The results of the MTT assay demonstrated that both 7CrHAp and 7CrHAp-CH coatings maintained a good biocompatibility with MG63 cells for both tested incubation times. For the 7CrHAp coatings, cell viability values remained above 90%, reaching approximately 91% at 24 h and increasing slightly to 92% after 48 h, suggesting that the coating surface supports the proliferation of osteoblast-like cells. Similarly, the 7CrHAp-CH coatings also showed high biocompatibility, with cell viability consistently above 91%. The values were close to 91% at 24 h and increased to 93% at 48 h, in line with the requirements of ISO 10993-1:2018 [52], which defines materials with viability above 88% as biocompatible. These findings indicate that both coatings did not exert significant cytotoxic effects on MG63 cells during the 24–48 h incubation period. The slightly higher cell viability values observed after 48 h further confirm the favorable biological response and suggest that 7CrHAp and 7CrHAp-CH coatings can provide a stable and supportive surface for cell adhesion and proliferation in biomedical applications.

**Figure 18:**
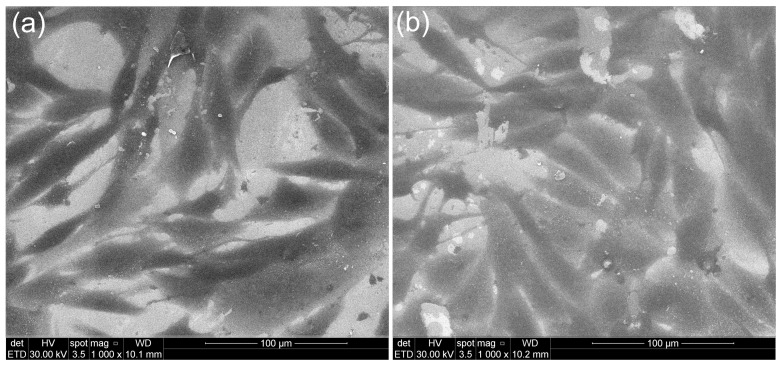
SEM micrographs of MG63 cells attachment on the surface of 7CrHAp (**a**) and 7CrHAp-CH (**b**) coatings after 48 h incubation.

Complementary information regarding the MG63 cells’ adhesion and development on the surface of 7CrHAp and 7CrHAp-CH coatings was obtained through SEM visualization. After 48 h of incubation, the adhered MG63 cells were fixed on the surface coatings with the aid of glutaraldehyde, dehydrated with ethanol, and air-dried prior to SEM examination. The obtained micrographs are presented in Figure 18.

Studying the interaction between osteosarcoma MG63 cells and the 7CrHAp and 7CrHAp-CH coatings is essential for assessing their potential in biomedical applications. SEM represents a valuable tool in this context, providing detailed insights into cell–surface interactions at the microstructural level, including adhesion, spreading, and morphology. The SEM observations depicted in Figure 18a revealed that MG63 cells cultured on the 7CrHAp surface adhered well, firmly attaching to the coating and exhibiting a healthy morphology having feature representative of MG63 cells. Moreover, the SEM micrographs of the 7CrHAp-CH coating presented in Figure 18b showed an even more pronounced cell adherence. The cells displayed more extensive filopodia and lamellipodia, which were widely spread across the coating surface, suggesting improved anchorage and the presence of an enhanced interaction between the cells and the surface coating compared to 7CrHAp. These structures, essential for cell adhesion and intracellular signaling, indicate that the chitosan-modified coating provides a more favorable microenvironment for MG63 attachment and spreading. In addition, on both 7CrHAp and 7CrHAp-CH coatings, the adhered cells exhibited a flattened morphology with widespread cytoplasmic extensions, confirming robust and healthy adhesion. However, the SEM results highlighted that MG63 cells spread more extensively on the 7CrHAp-CH surface, emphasizing the improved biocompatibility and osteoconductive potential imparted by the chitosan incorporation. Thus, the SEM findings demonstrate superior MG63 cell adherence on the 7CrHAp-CH coating compared to 7CrHAp. These observations, together with the complementary MTT assay results, support the potential of these coatings in biomedical applications, such as implant surface modifications and bone graft substitutes. Furthermore, the results underline the advantage of combining chromium-doped apatite with chitosan to optimize biological performance.

**Figure 19:**
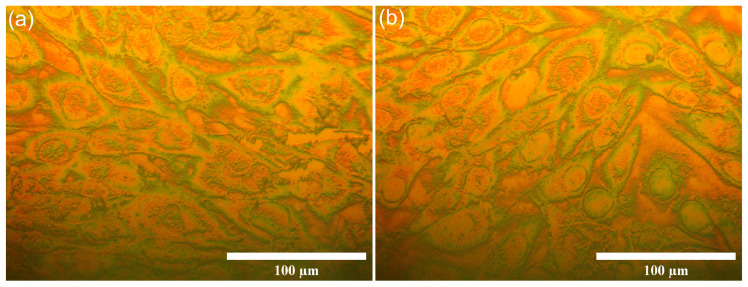
The morphology of MG63 cells grown on the 7CrHAp coatings (**a**) and 7CrHAp-CH coatings (**b**) visualized by metallographic microscopy (50X) after 48h of incubation.

Additional information regarding the MG63 cell development and adherence on the 7CrHAp and 7CrHAp-CH coatings after 48 h incubation were obtained through metallographic microscopy visualization. This technique was used to evaluate the adhesion and development of MG63 cells on the coating surfaces. The microscopy observations, illustrated in Figure 19, revealed that both 7CrHAp and 7CrHAp-CH coatings effectively supported MG63 cell adhesion and growth.

The adhered cells exhibited a normal morphology representative of normal MG63 cells, with no detectable abnormalities. These findings are consistent with literature reports [19,53,54], confirming that MG63 cell viability improves with prolonged incubation (24–48 h) on Cr-based hydroxyapatite coatings. The positive increase in cell viability over time emphasizes that the biocompatibility and bioactivity of the 7CrHAp and 7CrHAp-CH coatings, as well as their ability to sustain cell attachment, proliferation, and early differentiation, are kept in time. Although the precise molecular mechanisms that are involved in the interactions between the cells and the surface coatings is not yet fully elucidated, the current results clearly indicate a favorable biological response of MG63 cells to both 7CrHAp and 7CrHAp-CH coatings making them possible candidates in the future development of coatings for biomedical applications.

**Figure 20:**
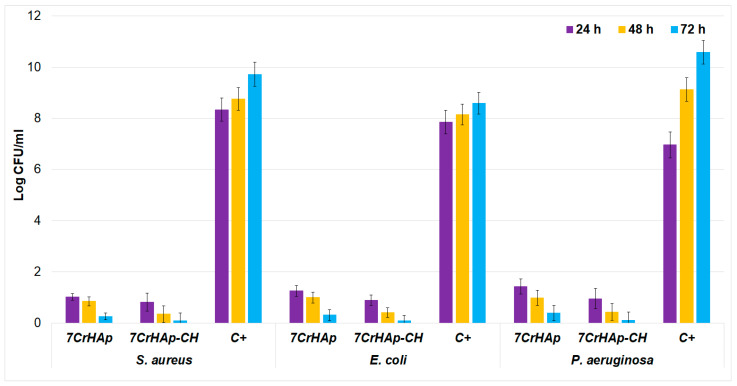
Graphical representation of the log colony forming units (CFUs)/mL of the 7CrHAp and 7CrHAp-CH coatings incubated with *Pseudomonas aeruginosa* 27853 ATCC, *Staphylococcus aureus* ATCC 25923 and *Escherichia coli* ATCC 25922 for 24, 48, and 72 h. The results were represented as mean values ± standard deviation (mean ± SD).

The antibacterial properties of 7CrHAp and 7CrHAp-CH coatings, was evaluated in vitro against one of the most common Gram-negative bacterium, *Pseudomonas aeruginosa*, which is associated with severe infections in the blood, lungs, and other organs. Furthermore, the antibacterial activity of the 7CrHAp and 7CrHAp-CH was also evaluated against Gram-positive, *Staphylococcus aureus* ATCC 25923 and Gram-negative strain *Escherichia coli* ATCC 25922. The antibacterial activity of 7CrHAp and 7CrHAp-CH coatings were assessed at three different time intervals. The results are presented graphically in Figure 20 as mean ± SD.

The results showed of the in vitro antibacterial assays revealed a significant reduction in the colony-forming units (CFUs) of *P. aeruginosa* after 24, 48, and 72 h of exposure to the 7CrHAp and 7CrHAp-CH coatings. The quantitative analysis highlighted that the 7CrHAp-CH coatings antibacterial effects was more pronounced, producing a greater decrease in CFUs compared to the control and 7CrHAp coatings alone. The in vitro quantitative assays also highlighted that the antibacterial activity of the coatings was also influenced by the incubation time. The reduction in colony-forming units (CFUs) became more pronounced for both types of coatings as the incubation time increased. This enhanced antibacterial effect over time may be attributed to the gradual and sustained release of chromium ions from the coatings, which likely contributes to the progressive inhibition of the bacterial cell’s growth. The antibacterial action is attributed to chromium ions release that is known for having the ability to disrupt bacterial membranes and metabolism, suggesting that metal ion release leads to a the time-dependent CFU reduction [55,56,57]. *Pseudomonas aeruginosa* has recently attracted a considerable attention due to its role as a major cause of healthcare-associated infections and also due to its notable resistance to antibiotics. More than that, *P. aeruginosa* can acquire resistance genes through horizontal gene transfer, further enhancing its survival against a wide range of antibiotics, including carbapenems, fluoroquinolones, and aminoglycosides. This multidrug resistance not only can complicate treatment strategies but also contribute to high morbidity and mortality rates, especially in the immunocompromised and hospitalized patients [58,59,60]. The enhanced antibacterial activity observed in the 7CrHAp-CH coatings could be attributed to the combined effects of chromium ions and chitosan. The synergy that appears in the biocomposite could be responsible for the superior inhibitory performance of 7CrHAp-CH against *P. aeruginosa* compared to 7CrHAp coatings.

The antibacterial assays clearly demonstrate that the incorporation of CH into 7CrHAp substantially enhances its antimicrobial performance against both Gram-positive (*S. aureus*) and Gram-negative (*E. coli* and *P. aeruginosa*) bacterial strain. The results of the in vitro antibacterial assay highlighted that 7CrHAp exhibited notable inhibitory effects, showing great reductions in the bacterial CFU after 72 h of incubation. More than that, the data also showed that 7CrHAp-CH almost completely eradicates the viable colonies across all the tested strains after 72 h of incubation. This highlights a strong synergistic effect between 7CrHAp and chitosan, suggesting that the addition of chitosan provides a significant advantage in suppressing the bacteria’s cells proliferation. The enhanced activity of 7CrHAp-CH may also be attributed to the intrinsic antibacterial properties of chitosan, which have been widely reported in the literature. Chitosan is known to exert its effects primarily through electrostatic interactions between its positively charged amino groups and negatively charged bacterial cell membranes, leading to disruption of membrane integrity, leakage of intracellular contents, and eventual cell death [61,62,63,64,65]. Furthermore, the time-dependent decrease in CFU values suggests that a sustained antibacterial activity is maintained over the tested time intervals, which is a desirable property for long-term biomedical applications such as coatings for implants or scaffolds for tissue engineering. Notably, *P. aeruginosa*, a pathogen who is notorious for its intrinsic resistance to antimicrobial agents and its biofilm-forming capacity [63], was also significantly inhibited by 7CrHAp-CH. The ability of 7CrHAp-CH to suppress this pathogen highlights its potential utility in preventing infections that are otherwise difficult to manage. Overall, these findings highlight the potential of 7CrHAp-CH as a promising antibacterial biomaterial. By combining the biocompatibility and osteoconductive properties of hydroxyapatite with the potent antibacterial activity of chromium ions and chitosan, the composite material not only inhibits a broad spectrum of bacterial strains but also has the ability to maintain a long-term antibacterial activity.

The results presented in this manuscript are in good agreement with previous reported studies and demonstrate that the reduction in CFU observed in the presence of 7CrHAp and 7CrHAp-CH is mainly attributable to the gradual release of chromium ions, combined with the bioactive effects of chitosan. As previously discussed, chitosan can directly impair bacterial division and growth, rather than merely slowing the growth rate. Our experiments, conducted at 24, 48, and 72 h, capture the sustained impact of these agents on bacterial viability. These findings are consistent with earlier studies showing that *Pseudomonas aeruginosa* control samples display a classical sigmoidal growth curve, with rapid CFU increases during the logarithmic (log) [66]. Other reports indicate that *P. aeruginosa* continues to accumulate biomass, with CFU counts increasing by approximately 1 log over 48 h, highlighting uninterrupted cell growth beyond the initial phase [67]. Similarly, in more natural environments such as mineral water, *P. aeruginosa* populations have been observed to rise by nearly 3 log units over 4–5 days before reaching a plateau [68]. Together, these studies and our results confirm that under control conditions, *P. aeruginosa* exhibits sustained CFU increases during the early to intermediate intervals (24–72 h), which are consistent with its characteristic growth kinetics. In contrast, the reduction in CFU in the 7CrHAp and 7CrHAp-CH groups reflects the well-established antimicrobial action of gradually released chromium ions, coupled with the bacteriostatic effect of chitosan, rather than being due to a generalized slowdown in bacterial growth.

Chitosan, well known as a polysaccharide produced by the deacetylation of chitin, has been reported to exhibit a broad-spectrum antibacterial activity against both Gram-positive and Gram-negative bacteria. These effects are primarily attributed to the electrostatic interactions between the positively charged amino groups (–NH_3_^+^) of chitosan and the negatively charged components of the bacterial cell walls. These interactions have the ability to increase the bacterial cell wall permeability, disrupting the cellular metabolism, and ultimately leading to bacterial cell death [69]. The antibacterial activity of chitosan has been highlighted to be stronger at lower pH, where it is more soluble and carries a higher positive charge, and more fable when the pH rises and solubility declines [70]. Furthermore, chitosan has also been reported to induce coagulation of bacterial proteins, which compromises membrane integrity, restricts nutrient access, and further disrupts cellular functions [60,61,62]. Chitosan also demonstrated notable antibacterial activity against *Staphylococcus aureus*, *Escherichia coli*, and *Pseudomonas aeruginosa*, although the degree of inhibition varied with the bacterial species [69,70,71,72,73,74,75]. As presented in previous reports, Gram-positive *S. aureus* appeared more susceptible, likely due to its exposed peptidoglycan layer facilitating electrostatic interaction with protonated amino groups of chitosan, leading to membrane disruption and leakage of intracellular contents [72]. In contrast, other studies reported that the Gram-negative bacteria exhibited higher tolerance, reflecting the protective role of their outer membrane; however, *E. coli* and *P. aeruginosa* were still inhibited at moderate concentrations, in line with findings that chitosan can destabilize lipopolysaccharide layers and increase membrane permeability [73]. Notably, *P. aeruginosa*, which is a highly resistant pathogen with robust biofilm-forming ability, has been reported to be inhibited at concentrations as low as 32 µg/mL, and chitosan has also been reported to act synergistically with conventional antibiotics against this species [74,75]. These results highlight that beyond direct bactericidal activity, chitosan may serve as an adjuvant, enhancing antibiotic efficacy and disrupting biofilm matrices [70]. Taken together, the evidence supports chitosan’s potential as a versatile antimicrobial agent, with particular promise for applications where biofilm control and combination therapy are desirable.

**Figure 21:**
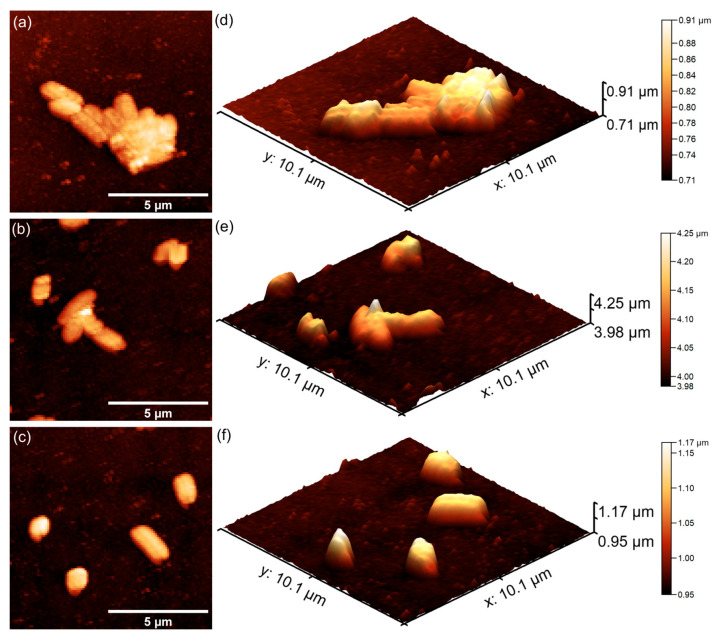
Two-dimensional AFM topography images of *Pseudomonas aeruginosa* ATCC 27853 cells adhered to the surface of 7CrHAp coatings after 24 (**a**), 48 (**b**), and 72 h (**c**) of incubation, along with their corresponding 3D representations (**d**–**f**).

**Figure 22:**
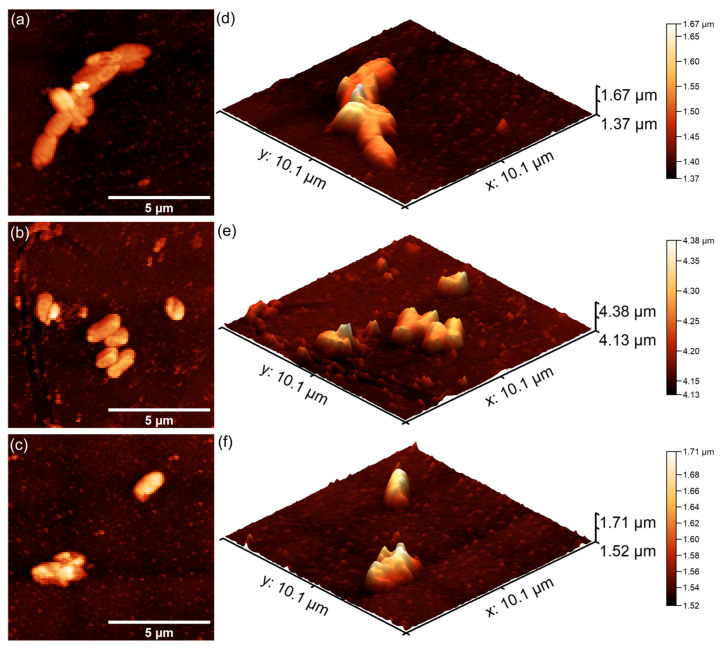
Two-dimensional AFM topography images of *Pseudomonas aeruginosa* ATCC 27853 cells adhered to the surface of 7CrHAp-CH coatings after 24 (**a**), 48 (**b**), and 72 h (**c**) of incubation, along with their corresponding 3D representations (**d**–**f**).

Furthermore, additional studies were conducted in order to investigate the adhesion and proliferation of *P. aeruginosa*, *E. coli* and *S. aureus*, on 7CrHAp and 7CrHAp-CH coatings with the aid of Atomic Force Microscopy (AFM). AFM topographies of the coatings after being incubated for three different time intervals with *P. aeruginosa*, *E. coli* and *S. aureus* bacterial suspensions were recorded in order to assess the antibacterial roles of chromium ions and chitosan. The AFM studies were conducted after incubating the coatings with *P. aeruginosa*, *E. coli* and *S. aureus* suspensions for 24, 48, and 72 h under ambient conditions and room temperature. Two-dimensional (2D) surface topographies were acquired in non-contact mode over areas of 10.1 × 10.1 µm^2^. Both 2D and three-dimensional (3D) AFM images of the 7CrHAp and 7CrHAp-CH surfaces are shown in Figure 21 and Figure 22.

The AFM studies demonstrated that both 7CrHAp and 7CrHAp-CH coatings effectively inhibited the initial adhesion and growth of *P. aeruginosa* cells. More than that, the AFM results also highlighted that the coatings inhibited *P. aeruginosa* bacterial cell’s biofilm formation. The 2D AFM images showed that the adhered bacterial cells exhibited a characteristic rod-shaped morphology, having 1.06–2.67 µm in length and 0.61–0.91 µm in width. Compared to 7CrHAp, the CrHAp-CH coatings exhibited enhanced antibacterial performance, suggesting a synergistic interaction between chromium ions and chitosan. The results of the AFM studies revealed that a significant reduction in bacterial attachment on the coating’s surfaces was observed within the first 24 h of incubation. Moreover, the images highlighted that the number of adhered bacterial cells significantly decreased with the increase in the incubation time. After 72 h, only a few isolated bacterial cells could be observed on the surface of7CrHAp and 7CrHAp-CH coatings, as verified through both 2D and 3D AFM imaging.

**Figure 23:**
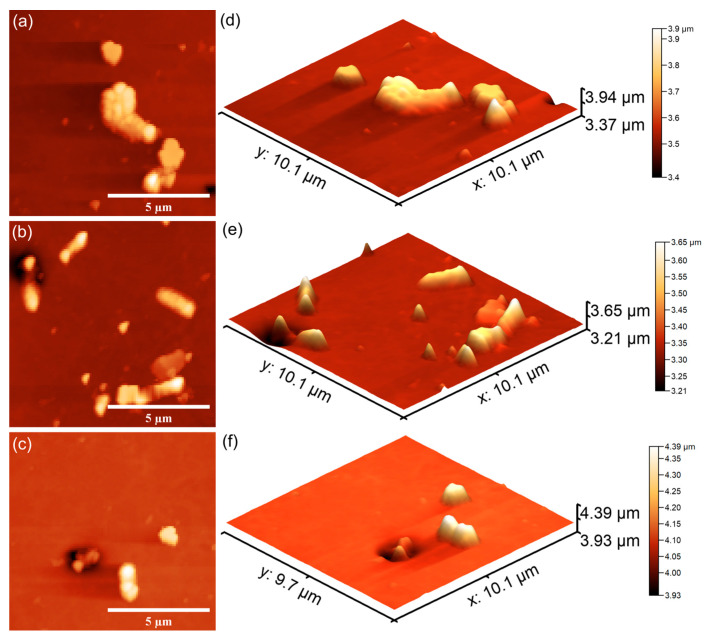
Two-dimensional AFM topography images of *Escherichia coli* ATCC 2592 cells adhered to the surface of 7CrHAp coatings after 24 (**a**), 48 (**b**), and 72 h (**c**) of incubation, along with their corresponding 3D representations (**d**–**f**).

**Figure 24:**
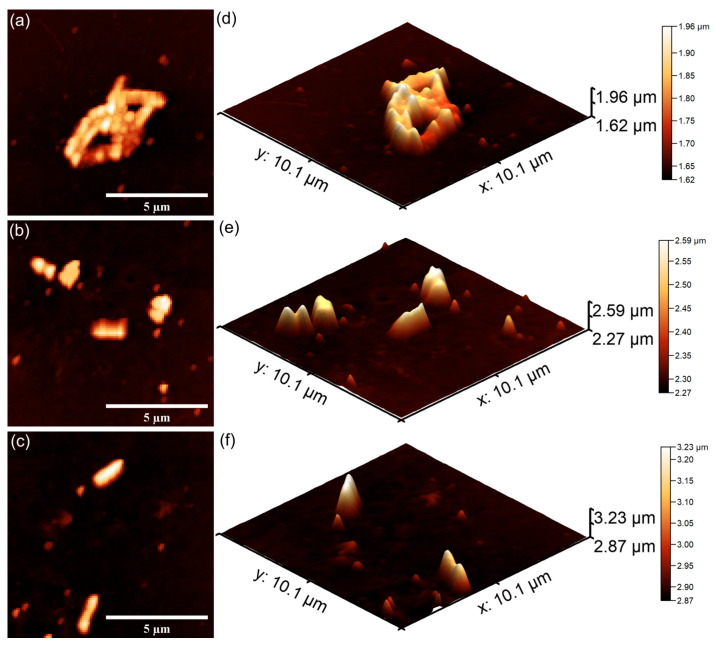
Two-dimensional AFM topography images of *Escherichia coli* ATCC 2592 cells adhered to the surface of 7CrHAp-CH coatings after 24 (**a**), 48 (**b**), and 72 h (**c**) of incubation, along with their corresponding 3D representations (**d**–**f**).

Furthermore, the results of the AFM investigation regarding the antibacterial effects of 7CrHAp and 7CrHAp-CH against *E. coli* bacterial cells adherence and proliferation, depicted in Figure 23 and Figure 24 also emphasize that the coatings exhibited good antibacterial effects even from the early adherence phase (the first 24 h). More than that, the AFM topographies reveal that the coatings did not allow the development of bacterial biofilm on the investigated coatings for any of the tested time intervals. The 2D AFM topographies confirmed that the adhered *E. coli* cells retained their characteristic rod-shaped morphology, having lengths ranging from 1.21 to 2.89 µm and widths from 0.53 to 0.96 µm [65]. Furthermore, both the 2D AFM topographies as well as their 3D representations emphasized that the 7CrHAp-CH coatings exhibited a better antibacterial activity compared to the 7CrHAp coatings, which could be attributed to a synergistic interaction between chromium ions and the chitosan matrix [72,74,75].

The AFM images also showed that the number of *E. coli* adhered bacterial cells to the surface decreased with the increase in the incubation time. After 72 h, only a few scattered bacterial cells could still be seen on the surfaces of both 7CrHAp and 7CrHAp-CH coatings. This observation was supported by both two-dimensional and three-dimensional AFM imaging, which confirmed the notable decrease in bacterial cell attachment over time.

**Figure 25:**
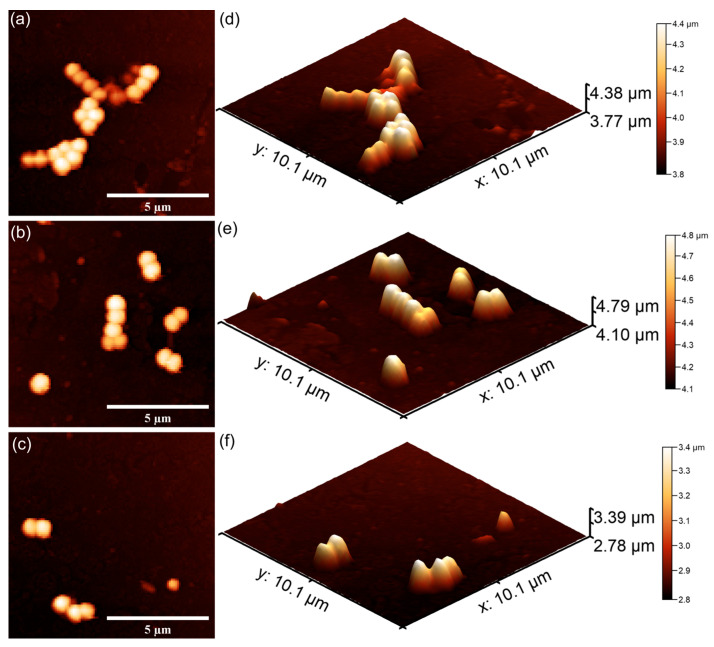
Two-dimensional AFM topography images of *Staphylococcus aureus* ATCC 25923 cells adhered to the surface of 7CrHAp coatings after 24 (**a**), 48 (**b**), and 72 h (**c**) of incubation, along with their corresponding 3D representations (**d**–**f**).

**Figure 26:**
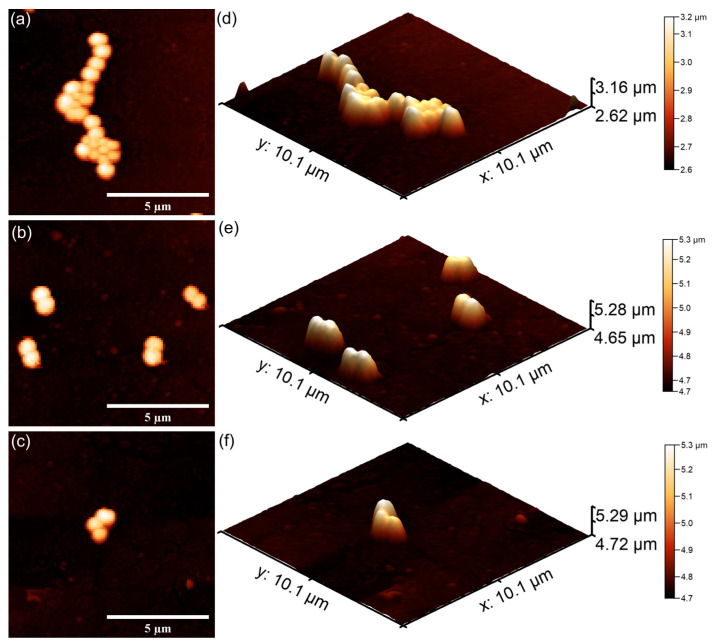
Two-dimensional AFM topography images of *Staphylococcus aureus* ATCC 25923 cells adhered to the surface of 7CrHAp-CH coatings after 24 (**a**), 48 (**b**), and 72 h (**c**) of incubation, along with their corresponding 3D representations (**d**–**f**).

The AFM topographies depicting the *S. aureus* bacterial cells adhered on the surfaces of 7CrHAp and 7CrHAp-CH coatings are presented in Figure 25 and Figure 26. The 2D AFM topographies as well as their 3D representation revealed that the coatings were effective in inhibiting *S. aureus* biofilm formation on their surfaces. More than that, the AFM analysis revealed that the coatings exhibited a strong inhibitory effect against *S. aureus* bacterial cells from the early adherence phase in the first 24 h. Additionally, the 2D AFM topographies confirmed that the adhered *S. aureus* cells adhered to the 7CrHAp and 7CrHAp-CH surface coatings retained the typical characteristic features of *S. aureus* bacterial cells exhibiting a spherical morphology, with diameters ranging from 0.41 to 0.84 µm [64]. Furthermore, the AFM studies suggested that the antibacterial activity increases gradually with increase in the incubation time.

The results of the AFM analysis demonstrated that the number of *S. aureus* cells adhered to the surface gradually decreased with the increase in the incubation time. After a period of 72 h, only a few scattered cells were detected on both 7CrHAp and 7CrHAp-CH coatings. This reduction was evidenced by both 2D and 3DAFM images, which confirmed the significant decrease in bacterial attachment. Considering the well-documented ability of *S. aureus* to colonize biomaterials through surface adhesions and to establish biofilms via polysaccharide intercellular adhesion and extracellular polymeric substances, the observed decrease in the bacterial cells attachment on the coatings’ surface suggests that these coatings may effectively inhibit the early adhesion events and also stop biofilm maturation. Such inhibition is of particular importance since *S. aureus* biofilms are typically associated with persistent infections and increased resistance to host immune responses and antibiotics [64]. The findings therefore indicate that 7CrHAp and 7CrHAp-CH coatings possess promising anti-adhesive properties against *S. aureus* bacterial cells making them possible good candidates for future development of antibacterial coatings for biomedical applications.

These findings demonstrate that the coatings efficiently prevent bacterial colonization and biofilm formation over extended periods. These antibacterial properties are particularly beneficial in medical fields where implantable devices, such as bone grafts, prosthetics, or dental implants, are at risk of microbial colonization and biofilm formation. Additionally, these materials could be valuable in wound care applications, where infection control is vital to successful healing and recovery. More than that, the antibacterial properties of the 7CrHAp-CH coatings and 7CrHAp compared to the control could be attributed both to chromium ions and the superior antibacterial activity exhibited by the 7CrHAp-CH coatings may be attributed to complementary mechanisms of chromium ions and chitosan that collectively contribute to bacterial cell death. The principal antibacterial mechanism reported for chromium ions is their ability to disrupt membrane integrity, leading to the loss of vital intracellular components such as ions, proteins, and nucleotides, and ultimately resulting in cell lysis [54,76]. Primarily, Cr^3+^ ions interact electrostatically with the negatively charged bacterial outer membrane of the bacterial cells, mainly lipopolysaccharides, compromising their membrane integrity and leading to an increased permeability and also to the leakage of intracellular contents [54,76]. Also, another mechanism responsible for the antibacterial properties is their ability to stimulate the production of reactive oxygen species (ROS), which cause oxidative damage to key cellular structures, including DNA, proteins, and lipids. This oxidative damage impairs the normal functioning of these molecules, leading to genetic mutations, protein denaturation, and membrane lipid peroxidation, further compromising cell viability. While the membrane disruption and oxidative stress induced by chromium ions are well-documented and reported in numerous studies, the full spectrum of antibacterial mechanisms of Cr^3+^ ions have yet to be completely elucidated. Ongoing research continues to study how these ions interact with bacterial cells on a molecular level, including their potential to interfere with metabolic pathways, enzyme activities, and signal transduction processes. Understanding these mechanisms in greater detail could provide valuable insights for the development of chromium-based antimicrobial strategies [54,76,77,78]. In addition, chitosan, which is a natural biopolymer derived from chitin, exhibits antibacterial properties through several distinct mechanism. Primarily, chitosan could bind to the negatively charged bacterial membranes via electrostatic interactions, increasing the membrane permeability and causing leakage of its intracellular contents. Moreover, chitosan has also been reported to be able to penetrate the bacterial cell wall and interact with DNA, therefore leading to an inhibition of transcription and protein synthesis. Moreover, chitosan also chelates with essential metal ions, thus disrupting the enzymatic systems and impairing microbial metabolism [72,79,80,81,82,83]. Its film-forming ability further contributes by creating a physical barrier that restricts nutrient access and bacterial adhesion [72,79,80,81,82,83]. Therefore, the combined antibacterial effects of chromium ions and chitosan exhibit a complex antibacterial effect that might include antibacterial mechanism such as membrane destabilization, oxidative stress, genetic interference, and metabolic disruption. These synergistic mechanisms align well with previously reported studies and support the development of durable, long-lasting antibacterial coatings.

In addition, the results of the in vitro quantitative and qualitative antibacterial assays highlighted that 7CrHAp exhibited an intrinsic antibacterial activity attributed to the presence of chromium ions, while 7CrHAp-CH exhibited direct antibacterial effects due to chitosan’s ability to disrupt bacterial cell membranes and inhibit microbial growth. Comparative antibacterial studies showed that 7CrHAp-CH coatings demonstrate the greatest CFU reduction compared to control. The superior antibacterial effects of 7CrHAp-CH coatings result from a synergistic effect of both 7CrHAp and chitosan, combining the bioactive properties of 7CrHAp with the natural antimicrobial activity of chitosan. Additionally, the results also emphasize that 7CrHAp-CH coatings could be considered for being used in medical devices, implants, and hospital surfaces to inhibit bacterial colonization and biofilm formation more effectively than other single-component coatings.

The present study makes a major contribution to the development of materials that could be successfully used for the production of coatings for various surfaces, medical devices, hospital surfaces, etc. These coatings can lead to the inhibition of bacterial colonization and the decrease in postoperative risks. The use of Si disks (which mimic the smooth surface of some medical implants) as a substrate provided a model system for studying the performance of the coating before application on more complex geometries. These coatings on Si disks were mainly used for in vitro testing of biocompatibility, antimicrobial efficacy and physicochemical properties, with the aim of developing safer and more infection-resistant biomaterials for bone and dental implants. It is known that both chromium-doped hydroxyapatite and chitosan exhibit antimicrobial properties. Thus, the use of Si disks as a substrate allowed the efficient evaluation of 7CrHAp and 7CrHAp-CH materials against bacteria (e.g., *Pseudomonas aeruginosa*), which is crucial for the prevention of implant-associated infections.

Furthermore, the deposition of four consecutive layers of 7CrHAp and 7CrHAp-CH on silicon (Si) disks offers several advantages, especially in biomedical research and application development. Also, by depositing multiple layers, the reproducibility of the materials can be improved from both a physicochemical and biological point of view. Thus, multiple layers increase the total thickness, which can improve mechanical stability, durability, and resistance to wear. Consecutive deposition helps to achieve a more uniform and defect-free coating, reducing holes or weak spots that could compromise performance. Another advantage of the successive layering was the increase in the concentration of chromium and chitosan, enhancing the antibacterial effects. As observed in this study, the multilayer coating contributed to the gradual release of bioactive ions (e.g., Ca^2+^, Cr^3+^) from the deeper layers, prolonging the antimicrobial effects.

AFM analysis confirmed the presence of chitosan in the 7CrHAp-CH sample, consistent with findings from XPS measurements. AFM analysis showed that the 7CrHAp-CH sample exhibited a lower root mean square (R_RMS_) roughness compared to the 7CrHAp sample. This observation aligns with XRD results, which indicate a smaller crystallite size for the chitosan-containing sample. The reduced surface roughness can be attributed to both the finer crystallite structure and the presence of chitosan, which functions as a binder and filler—effectively smoothing the surface by embedding or coating the hydroxyapatite particles [40,44,45,46,47,84]. Therefore, in the case of chromium-doped hydroxyapatite in a chitosan matrix, the smaller crystallite size probably contributes to a smoother surface profile—hence the lower RMS roughness observed by AFM.

The spectral change observed in the XPS spectra of the 7CrHAp and 7CrHAp-CH samples is a strong indicator of surface modification, which could influence properties such as biocompatibility, drug loading or ion exchange behavior. The deposition of four consecutive layers of CrHAp and 7CrHAp-CH on Si disks improved the coating thickness, uniformity, functional properties (antimicrobial and biocompatibility). We believe that this approach is particularly valuable for the development of advanced biomaterials for orthopedic and dental implants, where resistance to infection and tissue integration are essential.

## 4. Conclusions

Using stable suspensions of 7CrHAp and 7CrHAp-CH obtained by an adapted sol–gel method, uniform and homogeneous coatings could be prepared by dip coating. Cr^3+^ ions were successfully incorporated into the HAp lattice. Also, the chromium ion-doped hydroxyapatite was uniformly embedded in the chitosan matrix. The results of the FTIR studies revealed the presence of the functional groups specific to HAp and chitosan in the 7CrHAp-CH.

The in vitro antibacterial activity of the 7CrHAp and 7CrHAp-CH coatings against *Pseudomonas aeruginosa* was evaluated and confirmed through standardized antibacterial assays. The results of the antibacterial tests demonstrated that both 7CrHAp and 7CrHAp-CH coatings exhibited excellent antibacterial properties. The findings also highlighted that 7CrHAp-CH showed a significantly higher inhibitory effect on *P. aeruginosa*, *E. coli*, and *S. aureus* compared to 7CrHAp, indicating that the presence of chitosan contributed to the enhancement of the antibacterial potential of 7CrHAp coatings. This enhanced activity may be attributed to the improved surface characteristics, chemical composition, or also to an increased ion release, all of which may contribute to bacterial membrane disruption or metabolic inhibition. Further complementary information for these findings was obtained by AFM analysis, which revealed a strong effect on the bacterial cells adhesion behavior when exposed to the coatings surface. These observations emphasized the strong antibacterial potential of the coatings, which is of critical importance for preventing infections associated with biomedical devices. The results of this study highlight 7CrHAp and 7CrHAp-CH coatings as promising multifunctional devices for biomedical applications. They effectively inhibit bacterial growth, making them strong candidates for the development of advanced coating systems that could be used in regenerative medicine and infection-resistant medical devices.

The in vitro biocompatibility assay highlighted that both types of coatings exhibited good biological properties. The MTT assay on MG63 cells demonstrated excellent biocompatibility, showing a high proportion of viable cells while the SEM and MM studies revealed that the cells adhered very well to the 7CrHAp and 7CrHAp-CH coatings surfaces and that the adhered cells exhibited a typical morphology of normal MG63. These findings suggest that the coatings can effectively support cell proliferation without exerting cytotoxic effects on MG63 cells and confirm their potential suitability for bone tissue engineering and implant applications, where compatibility with human osteoblast-like cells is essential.

However, this study has some limitations: (1) biological evaluations were limited to in vitro cell viability assays and do not fully predict the in vivo performance; (2) the long-term stability and degradation behavior of the coatings were not assessed; (3) antimicrobial activity was tested under limited conditions. Future studies should address these aspects to fully validate the potential of 7CrHAp and 7CrHAp-CH coatings for uses in biomedical applications.

## Figures and Tables

**Table 1:** Surface atomic composition (atomic %) of the 7CrHAp and 7CrHAp-CH materials.

Sample	C	O	Ca	P	Cr	N
7CrHAp	14.5	46.09	24.21	14.50	0.7	-
7CrHAp-CH	19.6	48.06	18.84	11.7	0.7	1.1

**Table 2:** XRD analysis results.

Sample	Lattice Parameter (Å)		c/a	Average Crystal Size (nm)	Unit Cell Volume (cm^3^)
	a-Axis	c-Axis			
7CrHAp	9.363	6.873	0.73	19.63	522
7CrHAp-CH	9.432	6.869	0.728	16.29	529

**Table 3:** Atomic composition (atomic %) of the 7CrHAp and 7CrHAp-CH materials obtained by EDS semiquantitative analysis.

Sample	O	Ca	P	Cr	N	C
7CrHAp	59.8	18.6	14.8	6.8	-	-
7CrHAp-CH	64.8	14	12.2	6.5	0.8	1.7

**Table 4:** The FTIR bands associated with the vibrational modes of the functional groups present in the 7CrHAp and 7CrHAp-CH samples.

7CrHAp FTIR Absorption Bands (cm^−1^)	7CrHAp-CH FTIR Absorption Bands (cm^−1^)	FTIR Absorption Bands Assignments
470, 564, 602, 962, 1031, 1096	468, 561, 600, 960, 1028, 1091	P – O/O – P – O vibrations in [PO_4_]^3−^
1424, 1456	1421, 1450,	C – O vibrations in [CO_3_]^2−^ (for the 7CrHAp); overlapped vibration of [CO_3_]^2−^ and C – H (for the 7CrHAp-CH)
-	1561	N – H vibrations (amide II)
1642	1637	O – H vibrations

## Data Availability

The original contributions presented in the study are included in the article, further inquiries can be directed to the corresponding authors.
